# The local molecular signature of human peripheral neuropathic pain

**DOI:** 10.1097/j.pain.0000000000003472

**Published:** 2024-11-25

**Authors:** Oliver P. Sandy-Hindmarch, Pao-Sheng Chang, Paulina S. Scheuren, Iara De Schoenmacker, Michèle Hubli, Constantinos Loizou, Stephan Wirth, Devendra Mahadevan, Akira Wiberg, Dominic Furniss, Margarita Calvo, David L.H. Bennett, Franziska Denk, Georgios Baskozos, Annina B. Schmid

**Affiliations:** a Nuffield Department of Clinical Neuroscience, John Radcliffe Hospital, University of Oxford, Oxford, United Kingdom; b Spinal Cord Injury Center, Balgrist University Hospital, University of Zurich, Zurich, Switzerland; c International Collaboration on Repair Discoveries, Faculty of Medicine, University of British Columbia, Vancouver, BC, Canada; d Oxford University Hospitals NHS Foundation Trust, Oxford, United Kingdom; e Balgrist University Hospital, University of Zurich, Zurich, Switzerland; f Royal Berkshire NHS Foundation Trust, Reading, United Kingdom; g Nuffield Department of Orthopaedics, Rheumatology and Musculoskeletal Sciences, University of Oxford, Oxford, United Kingdom; h Department of Physiology, Pontificia Universidad Católica de Chile, Santiago de Chile, Chile; i Wolfson Sensory, Pain and Regeneration Centre (SPaRC), King's College London, Guy's Campus, London, United Kingdom

**Keywords:** Morton's neuroma, Peripheral nerve injury, Neuropathic pain, Neuroinflammation, RNA sequencing, Immune system, Inflammation, Ligand-receptor analysis, Macrophages

## Abstract

Focal nerve injuries are often associated with neuropathic pain. Preclinical research suggests altered neuroimmune signalling underlies such neuropathic pain; however, its cause remains poorly understood in humans. In this multicentre cohort study, we describe the local cellular and molecular signature of neuropathic pain at the lesion site, using Morton's neuroma as a human model system of neuropathic pain (n = 22; 18 women) compared with nerves from participants without nerve injury (n = 11; 4 women). Immunofluorescent staining revealed demyelination and chronic infiltration of immune cells in Morton's neuroma. RNA bulk sequencing identified 3349 differentially expressed genes between Morton's neuroma and controls. Gene ontology enrichment analysis and weighted gene co-expression network analyses revealed modules specific for host defence and neurogenesis. Deconvolution analysis confirmed higher densities of macrophages and B cells in Morton's neuroma than control samples. Modules associated with defence response, neurogenesis, and muscle system development as well as macrophage cell populations identified by deconvolution correlated with patients' paroxysmal or evoked pain. Of note, we identified a consistently differentially expressed gene signature (*MARCO, CD163, STAB1*)*,* indicating the presence of a specific M(GC) subset of macrophages. *MARCO* gene expression correlated with paroxysmal pain. Targeted immunofluorescent analyses confirmed higher densities of intraneural CD163^+^MARCO^+^ macrophage subsets in Morton's neuroma. Our findings provide detailed insight into the local molecular signature in the context of human focal nerve injury. There is clear evidence for an ongoing role of the immune system in chronic peripheral neuropathic pain in humans, with macrophages and specifically the M(GC) MARCO^+^ subset implicated.

## Abstract 2

Supplemental Digital Content is Available in the Text.

Combining pain-profiling with RNA sequencing and immunohistochemistry of Morton's neuroma and noninjured nerves, we identify an ongoing role for intraneural inflammation in chronic peripheral neuropathic pain.

## 1. Introduction

Neuropathic pain is a debilitating condition that affects ∼9% of the population.^5^ Compared with pain of nociceptive origin, neuropathic pain is associated with higher pain severity,^58^ lower quality of life,^5,46^ and higher healthcare costs.^58^ The most common causes of neuropathic pain are peripheral neuropathies and, in particular, focal nerve injuries such as entrapment neuropathies.^56^ Focal nerve injuries are associated with both evoked and spontaneous neuropathic pain. Preclinical research suggests altered signalling between distinct cell types such as neurons, glia, and immune cells may underlie neuropathic pain.^14,17,19^ Transcriptomic analyses of human dorsal root ganglia associated with neuropathic pain caused by tumour lesions confirm a dominant role of neuroimmune mechanisms.^45,52^ However, the neuroimmune relationship at the site of the nerve lesion in the nerve trunk remains poorly understood in humans because of challenges associated with access to injured human nerves and a lack of modern anatomical and molecular techniques applied to human injured nerves. While some studies have focussed on a few selected genes using polymerase chain reaction methodology,^59^ 2 recent studies have used bulk RNA sequencing to characterise the transcriptomic signature in human distal nerve trunks. Ray et al.^51^ used postmortem tibial nerve tissues and identified sexually dimorphic expression of genes involved in pain, inflammation, and neuro-immunity. Welleford et al.^64^ used serial sural nerve biopsies to identify gene signatures associated with acute nerve repair following transection injury. They identified genes representing antiapoptotic signalling, neurotrophic factor processes, cell motility, and again immune cell chemotactic signalling to be differentially expressed. In both studies though, no clinical information was available on the presence or nature of neuropathic pain, thus precluding any inferences about the local molecular signature associated with the clinical features of neuropathic pain.

In this study, we used Morton's neuroma as a unique model system to study the local molecular signature at the site of nerve injury in the context of neuropathic pain. Despite its misleading name, Morton's neuroma is an entrapment neuropathy, in which a plantar digital nerve is compressed just proximal to its bifurcation under the transverse intermetatarsal ligaments of the foot.^3^ Patients develop typical neuropathic symptoms, including burning, paroxysmal pain, and paraesthesia.^1^ In some patients, treatment involves the surgical excision of the affected nerve, therefore offering a rare and unparalleled source of injured human tissue in the context of neuropathic pain. Using clinical phenotyping of neuropathic pain followed by immunohistochemical and transcriptomic analyses of Morton's neuroma compared with human control nerves, we aimed to describe the local cellular and molecular signature of neuropathic pain. Our histological findings indicate a demyelinating lesion with chronically heightened densities of intraneural immune cells, confirmed by the enrichment of gene signatures related to neurogenesis and the immune system. Macrophage populations identified by deconvolution analysis and a differentially expressed gene signature, characteristic of a specific M(GC) macrophage subset (*CD163*^*+*^*, MARCO*^*+*^*, STAB1*^*+*^), positively correlated with the severity of paroxysmal pain. Immunohistochemical analyses confirmed increased intraneural densities of CD163^+^MARCO^+^ macrophage persisting in the chronic stages of focal neuropathy.

## 2. Material and methods

### 2.1. Study design and participants

This study reports cross-sectional data from a prospective longitudinal multicentre cohort study. Twenty-two adults over the age of 18 years with clinically diagnosed Morton's neuroma who were listed for neuroma excision surgery were recruited from waiting lists of Oxford University Hospitals and Royal Berkshire NHS Foundation Trusts UK (n = 13) or Balgrist University Hospital, Zurich, Switzerland (n = 9) between March 13, 2019, and June 22, 2021 (interrupted by the COVID-19 pandemic). Patients were excluded if they had systemic diseases affecting neural tissues (eg, diabetic neuropathy), other neurological conditions affecting the lower limb (eg, lumbar radiculopathy), severe somatic diseases affecting the foot (eg, polyarthritis), previous extensive surgery of the forefoot which may have an impact on sensory function, cortisone injection for the operated Morton's neuroma within the preceding 3 months, or if they were pregnant. We also recruited 11 control participants from Oxford University Hospitals NHS Foundation Trust who underwent surgery in which unaffected neural tissue could be sampled. These included surgeries for nerve grafting, muscle flap, or lower limb amputations (eg, due to nonhealing fracture). Control participants did not have any current systemic or neurological disease and no clinical history of neuropathy affecting the excised control nerve. Ethical approval was received from the South Central - Oxford C Research Ethics Committee (REF 18/SC/0410), the London—Camden & Kings Cross Research Ethics Committee (REC16/LO/1920), and the Cantonal Ethics Committee Zurich (2018-02198). All participants provided informed written consent in accordance with the Declaration of Helsinki.

Whereas control participants provided demographic and medical history data on the day of surgery, patients with Morton's neuroma attended a preoperative session with an investigator.

### 2.2. Phenotypic data of the Morton's neuroma cohort

Age, sex, body mass index (BMI) and duration of Morton's neuroma symptoms (if applicable) were recorded for each participant. The severity of patients' symptoms was evaluated presurgery using a range of clinical questionnaires. The average severity of pain and numbness over the past 24 hours was recorded on separate visual analogue scales (VAS), where 0 indicated no symptoms and 10 indicated the worst symptoms imaginable. The severity and profile of neuropathic pain were evaluated with the Neuropathic Pain Symptom Inventory (NPSI),^8^ which is formed of 10 numerical rating scales from 0 (no pain) to 10 (worst pain imaginable). These scales are used to rate pain subtypes including burning, deep pressure pain, paraesthesia, paroxysmal symptoms, and evoked pain, as well as a composite score.

To determine the characteristics of the neuropathic pain profile of Morton's neuroma compared with other neuropathies, we compared NPSI data among people with Morton's neuroma (n = 56, of which n = 22 were included in the tissue analysis), spine-related leg pain (“sciatica,” n = 134), carpal tunnel syndrome (n = 113), and diabetic neuropathy (n = 628). The data for the latter 3 cohorts were available from our previously published studies (REC Ref 18/SC/0263 and 10/H0706/35).^6,54,57,61^

### 2.3. Tissue collection and preparation

From patients with Morton's neuroma, 22 affected plantar digital nerves were collected during routine surgical excision of the neuroma. Each Morton's neuroma sample was split into 4 pieces, 2 from the site of the neuroma just proximal to the bifurcation of the plantar digital nerve, and 2 just distal to the bifurcation (digital branches, Supplemental Figure A1, http://links.lww.com/PAIN/C177). One distal and one proximal sample were placed into RNAlater solution (ThermoFisher Scientific, Horsham, United Kingdom, AM7021) and stored overnight at 4°C. The remaining distal and proximal samples were placed in a 4% paraformaldehyde solution and stored at room temperature overnight. Only the proximal samples representing the site of Morton's neuroma were used in our analyses.

From control participants, 11 nerves of the upper and lower limbs were collected (5 nerve grafts, 3 from muscle flaps, 3 from amputations). Of these 11 control samples, 3 were plantar digital nerves, 3 were gracilis motor nerves, 4 were posterior interosseous sensory nerves, and 1 was an intercostal sensory nerve. Each sample was split into 2 samples and placed into RNAlater and paraformaldehyde as detailed above.

For molecular analyses, the proximal nerve samples stored in RNAlater solution had any extraneural connective tissue removed the next day. The samples were then snap frozen in an Eppendorf tube in liquid nitrogen and stored at −80°C for batch RNA extraction.

For immunofluorescent analysis, samples were placed in sucrose the next day for 3 days before being frozen in optimal cutting tissue gel and storage at −80°C.

### 2.4. Immunofluorescent analyses

Immunofluorescent staining was done on proximal nerve samples from patients with Morton's neuroma and control participants for general characterisation of the injury. We stained for the neuronal markers myelin basic protein (MBP) and β-tubulin to determine the extent of axonal loss and demyelination. We also evaluated intraneural infiltration of immune cells including macrophages and T cells and their specific subtypes. For each participant, 14 µm serial tissue sections were cut using a cryostat (Leica CM1860 UV, Wetzlar, Germany) and adhered to SuperFrost Plus microscope slides (VWR, 631-0108) coated with 0.01% poly-L lysine (PLL) solution (Sigma-Aldrich, Gillingham, United Kingdom, P8920). 400 µL of 0.01% PLL solution was added to each slide and left to dry at room temperature. The tissue sections were left to dry overnight at room temperature before being stored at −20°C for batch staining.

Before staining, the tissue sections were defrosted at room temperature for an hour and incubated at 60°C for another hour to improve tissue adherence. Then, mild heat-induced epitope retrieval (HIER) was applied for 5 hours by incubating the sections in 70°C Tris-EDTA buffer (pH 9.0) containing 10 mM Trizma Base (Sigma-Aldrich, T6066), 1 mM EDTA (Sigma-Aldrich, ED2SS) and 0.05% Tween-20 (Sigma-Aldrich, P7949) in distilled water. After HIER, the sections were washed once for 5 minutes in PBS-Tx: 0.2% Triton X-100 (Sigma-Aldrich, X100) in PBS. Then, the sections were incubated for an hour at room temperature in blocking solution containing 5% normal goat serum (NGS, Sigma-Aldrich, G6767), 1% bovine serum albumin (BSA, Europa Bioproducts Ltd., Borehamwood, United Kingdom, EQBAH65), 1% dimethyl sulfoxide (DMSO, Sigma-Aldrich, D5879), 0.5% skim milk powder (Sigma-Aldrich, 70166), 0.3% Triton X-100 and 0.1% NaN_3_ (Sigma-Aldrich, S8032) in PBS.

For evaluating the integrity of axons (β-tubulin III) and myelin sheaths (myelin basic protein [MBP]) and the density of immune cells of interest (CD68, CD3, CD4, MARCO, CD163), we double stained with the markers summarised in Supplemental Table A1, http://links.lww.com/PAIN/C177. The tissue sections were incubated with primary antibodies overnight at 4°C in a humidified chamber. The next day, sections were washed 5 times with PBS-Tx. Sections were then incubated with secondary antibodies for 2 hours at room temperature in a humidified chamber. Last, the tissue sections were washed 4 times with PBS-Tx and once with PBS before being mounted in Vectashield mounting media with DAPI (Vector Laboratories, Newark, NJ, H-1200). For CD163/MARCO staining, Nuclear Violet (AAT Bioquest, Pleasanton, CA, 17543, 1:1000) was used to visualise nuclei. Antibody validity was confirmed with positive controls (eg, human tonsillar tissue for immune cells), negative controls (eg, no-primary-antibody controls), and morphological plausibility (Supplemental Figure A2, http://links.lww.com/PAIN/C177).

The stained sections were imaged with an Observer Z1 confocal imaging system (Zeiss, Oberkochen, Germany) at 200-fold magnification, and maximum intensity projections were derived from Z-stacks. Depending on tissue area, 1-7 images (average 3) were taken per tissue sample for quantification purposes. The density of CD3^+^, CD68^+^, CD3^+^CD4^+^, CD163^+^, MARCO^+^, CD163^+^MARCO^+^ and CD163^+^MARCO^−^ cells, and the amount of β-tubulin III and MBP were quantified by an examiner blinded to group allocation using ImageJ (version 2.9.0). For β-tubulin III and MBP, intraneural areas were selected, and corrected mean gray values were determined by subtracting background mean gray values (average of 3 extraneural areas) from intraneural values. Area fractions were quantified as the percentage of intraneural pixels with positive signal (threshold = 30). Intraneural CD3^+^, CD68^+^, CD3^+^CD4^+^, MARCO^+^, CD163^+^, CD163^+^MARCO^+^, and CD163^+^MARCO^−^ cells were counted using the multipoint tool, and densities were established as cells/mm^3^. Only cells containing a Nuclear Violet positive nucleus were counted.

### 2.5. RNA sequencing

On the day of RNA extraction, 1 mL of TRIzol solution (ThermoFisher Scientific, 15596026) was added to the frozen sample which was then homogenised. Chloroform was added before centrifugation for phase separation. Total RNA extraction was done using a hybrid method of phenol extraction (TriPure; Roche, Welwyn Garden City, United Kingdom) combined with column purification (High Pure RNA Tissue Kit; Roche). The aqueous liquid phase containing the nucleic acids was removed and added to the columns of the High Pure RNA Tissue Kit (Roche Diagnostics). RNA was purified using repeated wash steps and DNAse treatment. The concentration of RNA in the samples was measured using a nanodrop. Total RNA was provided to the sequencing centre, and the poly-adenylated fraction was selected for sequencing.

For histological analyses, the samples stored overnight in paraformaldehyde solution were washed 3 times in 0.1M phosphate buffer and stored in a 20% w/v sucrose solution for 3 days at 4°C. Nerve samples were frozen in optimal cutting temperature gel in base moulds and frozen in liquid nitrogen. Samples were then stored at −80°C for immunofluorescence analyses.

RNAseq was performed at the Wellcome Centre for Human Genetics. All samples passed the initial QC assessing RNA degradation (RIN >8). Polyadenylated transcript enrichment and strand-specific library preparation were done using the NEBNext Ultra II RNA Library Prep kit (NEB, E7770) following the manufacturer's instructions. Libraries were amplified using unique dual indexing primers, based on Lamble et al.^28^ Paired-end sequencing was performed on an Illumina NovaSeq6000 system.

Quality control of the raw sequencing reads was performed using FastQC/MultiQC, assessing the yield, number and percentage of duplicate reads, the per sequence Phred quality score, the GC content, the length distribution, and overrepresenting sequences or adapter contaminations. Two of the sequenced libraries had an overrepresented sequence that indicated adapter contamination (1.38% and 3.27%). These were dealt by utilising the “soft clipping” technique of the aligner.

Reads were mapped to the GRCh38 human genome by the STAR aligner programme^13^ with standard ENCODE options, and gene counts were determined using HTSeq.^2^ The fraction of uniquely mapped reads across samples was excellent, median = 94.13% (IQR 93.45% - 94.52%), total number of pairs of reads median = 18953,721 (IQR 18165,457 – 20463,241). DESeq2^36^ was used to determine differential gene expression between nerve samples from patients with Morton's neuroma and control participants, controlling for sex effects by using an additive design of ∼ sex + condition. Moderation of log fold changes was carried out by using a zero-mean normal prior on the coefficients of interest. Gene ontology (GO) analyses were performed in R using the GOseq^66^ package. A probability weighting function was used to model gene length bias for the GO analysis, and the background consisted of all expressed genes with more than 20 counts in at least 50% of the samples. Genes with an false-discovery rate (FDR)-adjusted *P*-value < 0.05 and in the top 25% (third quantile) of log 2-fold changes (absolute LFC > 0.64) were considered significantly regulated for this and all downstream analyses. Unsupervised medoid “pam” clustering was used to identify groups of genes and samples with similar expression profiles. The optimal number of clusters was determined using the gap statistic. Hierarchical clustering was carried out using Ward method.

We then performed weighted gene co-expression network analysis (WGCNA) using the WGCNA^30^ package in R. Weighted gene co-expression network analysis is a method that builds a co-expression network based on the correlation between different genes and then identifies modules of highly correlated genes. An unsigned co-expression network was built using the top 25% of expressed genes (20 counts in at least 50% of samples) ranked by observed variance, and modules were identified using the dynamic tree cut algorithm.^29^ We assigned biological functions to these modules by looking at overrepresented GO biological process terms. We then used the module eigengene, ie, the first principal component of a given module, as the representative gene for each module to calculate associations with phenotypic traits. Deconvolution was done using the deconvolution pipelines from EPIC,^50^ quanTIseq,^16^ and GEDIT^43^ software packages and online tools. In the absence of reference sequences from human peripheral nerves, additional sequences were used from the ABIS^39^ and LM22^44^ datasets.

We also looked at the normalised expression pattern of genes transcribed in adult axons.^20^ We defined an “axonal signature” as the first principal component of the expression pattern of the axonally transcribed genes and looked at the coordinates of the projections of Morton's neuroma samples and control nerves.

We further used mouse macrophage profiling identified by sc-RNAseq at steady state and day 1 and day 5 post sciatic nerve crush^65^ combined with MuSiC2^63^ to estimate cell type proportions. We also carried out a ligand–receptor analysis taking into account the differential expression profiling in Morton's neuroma vs control nerves using BulkSignalR.^62^

### 2.6. Statistics

The sample size is based on a simulation of gene counts from our previous human RNAseq experiment in the skin of patients with and without nerve injury and neuropathic pain.^6^ A sample of n = 11 to 18 has 69 to 80% power to detect 10% differentially expressed genes assuming log-normal distribution of fold changes with mean log_2_1.5, a standard deviation 0.5 × log_2_1.5, and a sequencing depth of 24M 75 bp paired-end.

The statistical and graphical analyses were conducted using R,^49^ SPSS (version 28, IBM), and Prism 9 (version 9.5.1, GraphPad). Visual inspection as well as Shapiro–Wilk tests were used to test for normality within the data.

Neuropathic Pain Symptom Inventory data among different neuropathy cohorts were compared with a univariate analysis of co-variance, correcting for age and sex. Post hoc comparisons for each NPSI domain were corrected for 3 comparisons (Morton's neuroma vs “sciatica”/carpal tunnel syndrome/diabetic neuropathy) with *P*-values < 0.016 deemed significant.

For comparing the integrity of β-tubulin III and MBP as well as intraneural immune cell density between groups, independent sample Mann–Whitney *U* tests or Student *t* tests were applied with *P*-values < 0.05 deemed significant.

For RNAseq analyses, the Wald test was used to determine differences in gene expression with an FDR-adjusted *P*-value < 0.05 and an absolute log2 fold change > 0.64 deemed significant. This set of significantly regulated genes was considered for the GO enrichment analysis, which was performed separately for upregulated and downregulated genes. To determine the differences between deconvolution-derived cellular proportions, independent sample Mann–Whitney *U* tests were applied with an FDR-adjusted *P*-value < 0.05 deemed significant.

The correlations between WGCNA modules, their single genes or immunohistological data and patients' pain-related symptoms (NPSI subscores and VAS for numbness or pain) were evaluated using the Spearman rank correlation or Pearson correlation with an FDR-adjusted *P*-value < 0.05 considered significant.

### 2.7. Data availability

The RNA sequencing data that support the findings of this study are openly available in GEO following publication in a peer-reviewed journal (series number GSE250152). Data on clinical phenotypes of the Morton cohort are available from the corresponding author upon reasonable request. The data are not publicly available due to restrictions; for example, they contain information that could compromise the privacy of research participants.

## 3. Results

### 3.1. Demographic and clinical data of participants

Demographic and clinical data for study participants are shown in Table 1. Age, height, weight, and BMI were comparable, but the percentage of female participants was higher in the Morton's neuroma compared with the control group (82 vs 36%). Patients had on average a moderate severity of pain (median VAS 4.46 [IQR 4.42]). While the total NPSI scores were comparable among groups, the Morton's neuroma cohort reported more intense burning, deep, paroxysmal, and evoked pain than the carpal tunnel syndrome cohort (Supplemental Figure A3, http://links.lww.com/PAIN/C177). Evoked pain was also more intense in the Morton cohort compared with all other neuropathy cohorts. The self-reported sensory profiles of the Morton's neuroma cohort were otherwise largely comparable to the “sciatica” cohort, which represents another common entrapment neuropathy affecting the lower quadrant.

**Table 1:** Demographic and clinical data.

	Control	Morton's neuroma
No. of participants	11	22
Age (y)	58.0 [21.0]	60.0 [16.0]
Female sex, n (%)	4 (36.4%)	18 (81.8%)
Height (cm)	177.0 [18.0]	167.5 [12.0]*
Weight (kg)	78.0 [46.0]	70.0 [21.8]*
BMI (kg/m^2^)	24.9 [10.6]	24.9 [6.3]*
Duration of symptoms (mo)		30.0 [18.0]*
NPSI total score		30.0 [18.0]*
Burning pain		5.0 [8.0]*
Deep pain		3.0 [3.0]*
Evoked pain		3.5 [2.5]*
Paroxysmal pain		5.0 [7.0]*
Paraesthesia		2.0 [5.3]*
Visual analogue scale (average over past 24 h)		
Pain		4.5 [4.4]*
Numbness		0.6 [4.3]*

Data are presented as median [interquartile range] unless stated otherwise.

* Clinical data from 1 person with Morton's neuroma missing as questionnaires were not completed.

BMI, body mass index; NPSI, Neuropathic Pain Symptom Inventory.

### 3.2. Immunofluorescence reveals demyelination and T-cell infiltration in Morton's neuroma

**Figure 1.**
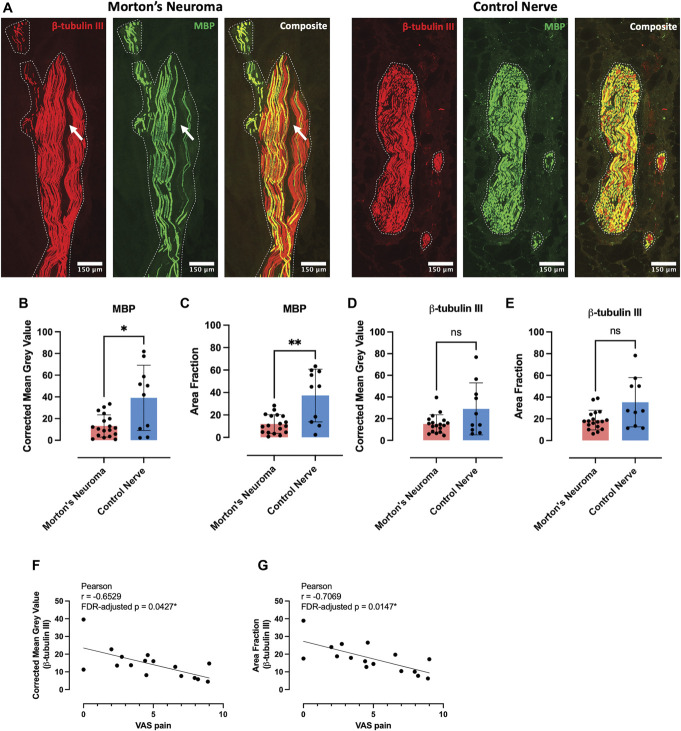
Immunofluorescence analysis reveals demyelination in Morton's neuroma. (A) Representative images (stitched tile scans with 5% overlap) of nerve sections from a patient with Morton's neuroma (left) and a healthy individual (right) stained with anti-β-tubulin III (red) and MBP (green). The boundaries of intraneural areas are indicated with white dotted lines. We repetitively identified several areas that are devoid of axons within the Morton's neuroma. One such region is identified in A with a white arrow. (B–C) Quantification revealed a reduction of MBP-corrected mean gray values (B) and area fractions (C) in Morton's neuroma compared with control nerves. (D–E) Corrected mean gray values and area fractions of β-tubulin III were comparable between groups. Morton's neuroma, N = 18; control nerve, N = 10. Data are presented as means and standard deviations. Student t tests, *P*-values = 0.024 (B), 0.008 (C), 0.106 (D), and 0.051 (E). (F) Corrected mean gray value and (G) area fraction of β-tubulin III staining strongly negatively correlated with patient's pain measured on a visual analogue scale (VAS). N = 16 (2 patients had missing VAS pain scores); Pearson correlation, FDR-adjusted *P*-values, *P* = 0.0427 and 0.0147, respectively. MBP, myelin basic protein.

We first used immunofluorescent analyses of longitudinal nerve sections to gain a general overview of axon and myelin integrity. Corrected mean gray values and area fractions of MBP were reduced in nerves from patients with Morton's neuroma compared with control nerves, indicative of demyelination (Figs. 1A–C). By contrast, there was no significant difference in β-tubulin III between groups (Figs. 1A, D–E). A negative correlation of β-tubulin III's area fraction and corrected mean gray value with the VAS for pain survived correction for multiple comparisons between axonal or myelination parameters with patients' pain phenotype (Figs. 1F–G, Supplemental Table A2, http://links.lww.com/PAIN/C177).

**Figure 2.**
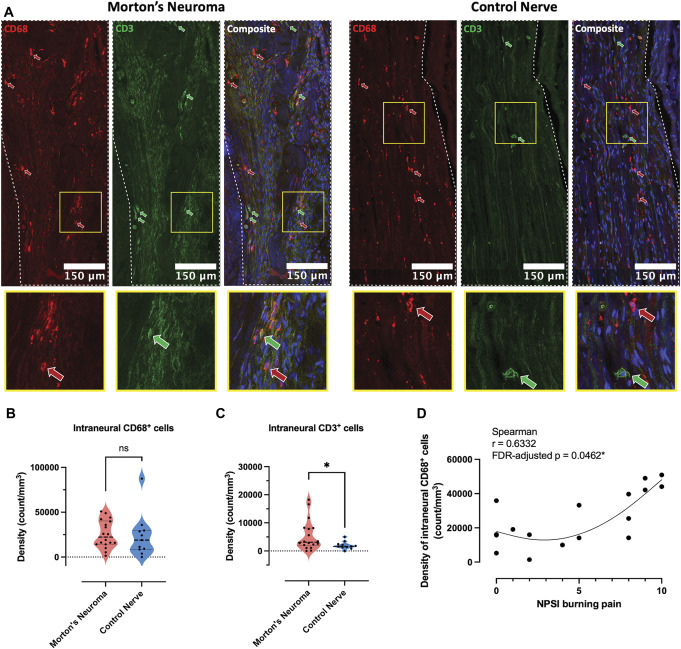
The density of intraneural CD3^+^ but not CD68^+^ cells is higher in patients with Morton's neuroma. (A) Representative images (stitched tile scans with 5% overlap) of nerve sections from a patient with Morton's neuroma (left) and a healthy individual (right) stained with CD68 (red) and CD3 (green). The boundaries of intraneural areas are indicated with white dotted lines. The areas within yellow squares were enlarged 7x and displayed below. The red and green arrows indicate examples of CD68^+^ and CD3^+^ cells, respectively. (B–C) Unlike CD68^+^ cells (B), the densities of intraneural CD3^+^ cells (C) were significantly higher in Morton's neuroma compared with control nerves. Morton's neuroma, N = 18; control nerve, N = 11. Data are presented as medians and interquartile ranges. Mann–Whitney *U* tests, *P*-values = 0.3397 and 0.0402, respectively. (D) The density of intraneural CD68^+^ cells was positively correlated with patients' burning pain measured on the Neuropathic Pain Symptom Inventory subscore, N = 18, Spearman correlation, FDR-adjusted *P* = 0.046.

We then used pan-macrophage and T-cell markers to determine immune cell infiltration. While there were no significant differences in the density of intraneural CD68^+^ cells between groups (Figs. 2A–C), the density of intraneural CD68^+^ cells significantly positively correlated with NPSI burning pain, (Fig. 2D, Supplemental Table A3, http://links.lww.com/PAIN/C177). There was a higher density of intraneural T cells in patients with Morton's neuroma compared with healthy controls (Figs. 2A, C), but densities did not correlate with patients' pain phenotype (Supplemental Table A3, http://links.lww.com/PAIN/C177). Further T-cell subtyping did not identify a significant difference in intraneural CD3^+^CD4^+^ cell densities between Morton's neuroma and control nerves (Supplemental Figure A4, http://links.lww.com/PAIN/C177) or their association with patients' phenotype (Supplemental Table A4, http://links.lww.com/PAIN/C177).

Finally, we found the area fractions of intraneural MBP, but not β-tubulin III, significantly negatively correlated with the densities of intraneural CD3^+^ and CD68^+^ cells in Morton's neuroma (Supplemental Table A5, Supplemental Figure A5, http://links.lww.com/PAIN/C177).

### 3.3. Differential gene expression analysis reveals a high level of differentially expressed genes

**Figure 3.**
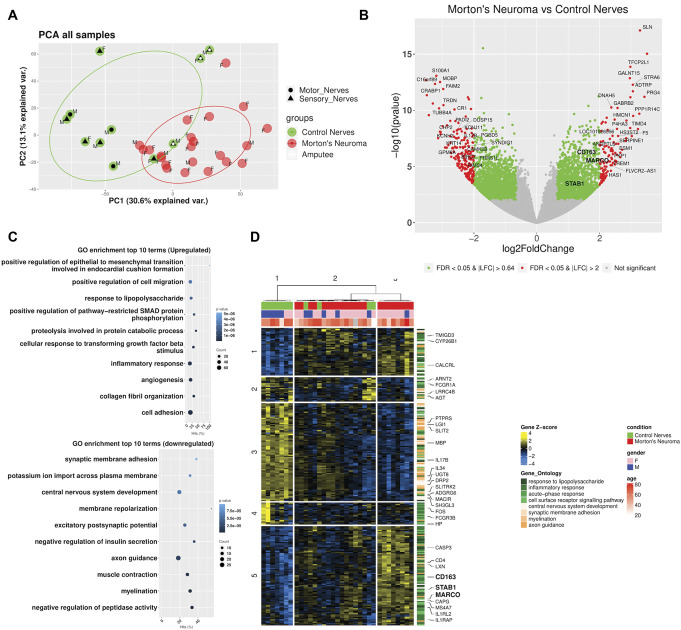
Differential gene expression analysis revealed a high number of differentially expressed genes between samples from patients with Morton's neuroma and control participants. (A) Principal component analysis plot showing good separation between patients with Morton's neuroma (red) and control participants (green). Control nerves from amputations are denoted by a blue dot; control motor and sensory nerves are denoted by a black circle or triangle, respectively. Circles represent the centroids and 95% CI of the respective groups. The first principal component accounted for 30.6% of the variance within the data, and the second principal component accounted for 13.1%. The sex of participants is indicated with M, men and F, women. (B) Volcano plot showing the level of differential gene expression. Genes shaded in red have an FDR-adjusted *P*-value < 0.05 and an absolute Log2FC > 2. Genes shaded in green have an FDR-adjusted *P*-value < 0.05 and an absolute Log2FC > 0.64, the top 25% quantile. Genes shaded in gray are nondifferentially expressed genes. (C) Dot plot of the top 15 enriched gene ontology terms ranked by their overrepresentation *P*-value for both upregulated and downregulated genes in Morton's neuroma vs controls. Hits (%) indicates the fraction of genes in the term that are differentially expressed. Counts indicate the number of genes in the term that are differentially expressed. (D) Heatmap showing the clustering of samples based on the expression levels of genes that belong in the top enriched biological processes associated with the response of the immune system and the development of the nervous system.

To gain a detailed understanding of the molecular signature associated with Morton's neuroma, we conducted differential gene expression analysis on the 22 plantar digital nerves from people with Morton's neuroma and the 11 control nerves. A principal component analysis (PCA) plot (Fig. 3A) shows good separation between the patient and control nerve samples. The majority of the variance can be attributed to principal component 1 (PC1), which accounts for 46% of the variance within the data, PC2 accounts for a further 13% of the variance. Differential gene expression analysis identified over 3349 genes to be differentially expressed (DEG, Fig. 3B): 1461 were upregulated and 1888 were downregulated. A large proportion of these genes had an absolute Log2 fold change (FC) of above 2. In line with our immunofluorescent findings, a GO enrichment analysis for the biological processes overrepresented showed that amongst genes upregulated in Morton's neuroma vs control nerves, there were significantly overrepresented terms related to the response of the immune system to inflammation including “response to lipopolysaccharide” and “inflammatory response,” Figure 3C. Amongst downregulated genes, there was a significant enrichment of processes related to the development of the nervous system including “axon guidance,” “myelination,” “synaptic membrane adhesion,” and “central nervous system development,” Figure 3C. A heatmap of the 238 differentially expressed genes that were annotated with the selected highly enriched terms related to the immune system and development of the nervous system (Fig. 3D), showed a mostly neuronal cluster of genes (cluster 3) downregulated in Morton's neuroma, 2 mixed clusters (clusters 1 and 2) with both inflammatory response and nervous system development related terms. A small cluster of immune response-related genes (cluster 4) was downregulated in Morton's neuroma, but the main cluster of inflammatory-related genes (cluster 5) was consistently upregulated in Morton's neuroma vs control nerves.

We found no differences in the axonal gene signature defined as the first principal component of their expression and no distinct expression pattern related to the Morton's neuroma vs control nerves (Supplemental Figure A6, http://links.lww.com/PAIN/C177).

A ligand-receptor (LR) analysis where LR interactions are inferred based on differential gene expression analysis in Morton's neuroma vs control nerves found that immune-related pathways, including cell surface interactions at the vascular wall, interleukin-4 and interleukin-13 signalling, interleukin-12 family signalling, IL-6-type cytokine receptor–ligand interactions, toll-like receptor cascades, and RAF/MAP kinase cascade, were enriched in the top pairs of the 32 significant LR interactors (Supplemental Table A6 and A7, http://links.lww.com/PAIN/C177). Moreover, the top LR pairs associated with these pathways include CD44 (marker of lymphocyte infiltration and M2 macrophage polarisation)^35,67^ and the genes ITGAM, CRLF1, ANGPTL1, HAS2, VCAN, SPON2, FN1, CLCF1 and TEK (Supplemental Figure A7, http://links.lww.com/PAIN/C177).

### 3.4. Immune and defence responses are overrepresented pathways

A WGCNA was conducted next to identify groups/modules of genes with similar expression patterns. We then determined overrepresented biological processes within these modules and looked for associations between the modules' representative genes (eigengene) and phenotypic traits.

**Figure 4.**
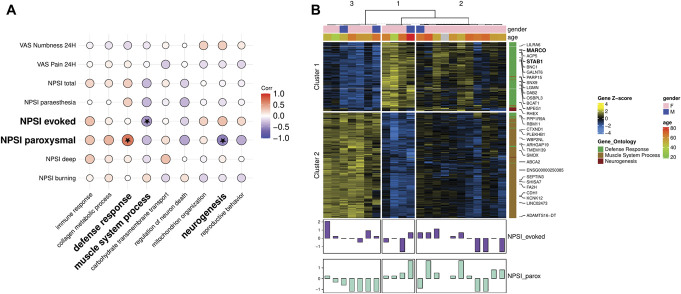
Weighted gene co-expression network analysis identifies modules associated with defence response and neurogenesis to be associated with patient's paroxysmal pain phenotype. (A) Correlation plot showing the Pearson correlation strength between phenotypic traits associated with the symptoms and modalities of pain and the eigengenes of the modules identified through the co-expression network analysis. The eigengenes are named after the top enriched biological process in the module. Correlation strength is encoded in the size and colour of the dots. Nonnominally significant associations (*P*-value > 0.05) are x crossed. (B) Heatmap showing the expression patterns and unsupervised clustering of samples based on the genes that belong in the modules that had significant correlations with phenotypic traits. The significantly correlated symptoms are z-transformed and shown in bar plots.

In the WGCNA analysis, we identified 9 modules (named after the top overrepresented GO term amongst the genes belonging to the module) that were enriched for the biological processes of immune and defence responses, regulation of neuron death and neurogenesis, collagen metabolism, muscle systems, carbohydrate transmembrane transport, mitochondrion organization, and reproductive behaviour. The eigengene of the module associated with defence response was positively correlated (r = 0.66, 95% CI [0.32, 0.85], *P*-value = 0.0007, FDR = 0.004) and the module eigengene associated with neurogenesis was negatively correlated (r = −0.54, 95% CI [−0.14, −0.79], *P*-value = 0.009, FDR = 0.05) with NPSI paroxysmal pain. The module eigengene associated with muscle system process was negatively correlated (r = −0.44, 95% CI [−0.01, −0.73], *P*-value = 0.03, FDR = 0.2) with NPSI evoked pain (Fig. 4A). These were moderate-to-strong correlations that all reached nominal significance, while the correlation of the defence response module eigengene with NPSI paroxysmal pain survived FDR adjustment. We then looked at the individual expression patterns of the top 25% genes belonging to these modules ranked by their correlation strength with the module eigengenes. We observed 1 group of Morton's neuroma patients with decreased expression in the defence response genes and lower paroxysmal pain; 1 group with higher expression in defence response genes and lower expression of muscle system process genes with higher paroxysmal and lower evoked pain; and finally, a third group with higher expression in neurogenesis genes and mixed expression of muscle process and defence response genes with a distinct anticorrelated expression pattern in the paroxysmal and evoked pain. Unsupervised clustering of genes revealed 2 clusters: cluster 1 had the genes in the modules associated with defence response and neurogenesis and cluster 2 had the genes associated with muscle system process (Fig. 4B, Supplemental File 1, http://links.lww.com/PAIN/C176).

### 3.5. Deconvolution analysis identifies significant differences in immune cell populations

**Figure 5.**
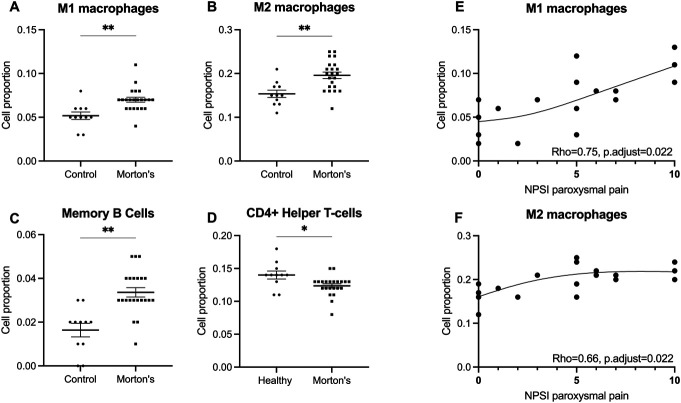
Deconvolution analysis reveals significant differences in immune cell proportions between samples from patients with Morton's neuroma and controls. (A–C) Macrophages and memory B-cell proportions were found to be significantly higher in samples from patients with Morton's neuroma compared with control participants. The package we used distinguished between macrophages according to the traditional M1 and M2 nomenclature. (D) CD4^+^ helper T-cell proportions were found to be significantly decreased in Morton's neuroma compared with control samples. (E, F) Both M1 and M2 macrophage proportions significantly positively correlate with NPSI paroxysmal pain. Significant differences between groups were determined by FDR-corrected Mann–Whitney U tests (adjusted *P* < 0.05). Spearman rank correlation was used with an FDR-adjusted *P* < 0.05. NPSI, Neuropathic Pain Symptom Inventory.

The large number of DEGs between Morton's neuroma and control nerves likely represents a change in, or recruitment of cell types, such as during an inflammatory or fibrotic response. Using deconvolution analysis to quantify immune cell proportions, we identified significant differences between samples of patients with Morton's neuroma and controls. Proportions of immune cell types including macrophages and memory B cells were higher in Morton's neuroma compared with control samples (Figs. 5A–C). The packages we applied to deconvolute used the traditional division of macrophages into M1 and M2, rather than updated nomenclature.^41^ Both types were increased in the neuroma, with the full list of genes denoted as “M1” and “M2” in the package provided in Supplemental Table A8, http://links.lww.com/PAIN/C177. CD4^+^ helper T cells were lower in patients with Morton's neuroma (Fig. 5D). Both M1 and M2 macrophage cell proportions positively correlated with the severity of paroxysmal pain (Figs. 5E and F). Integration with mouse single-cell data identified a macrophage subtype, ie, group 4 in Ydens et al.^65^ expressing Mgl2 and H2-Aa enriched on day 5 after experimental sciatic nerve injury in mouse was overexpressed in Morton's neuroma vs control nerves (Supplemental Figure A8, http://links.lww.com/PAIN/C177).

### 3.6. Genes specific to M(GC) MARCO^+^ subset of macrophages are upregulated and correlate with clinical phenotypes

**Figure 6.**
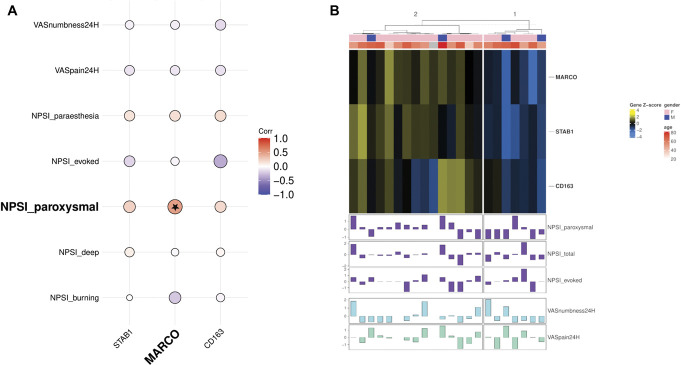
Individual genes representing an M(GC) MARCO^+^ subset of macrophages positively correlated with the paroxysmal pain phenotypes. (A) The expression of individual genes *MARCO, STAB1,* and *CD163*, denoting M(GC) macrophages, are positively correlated with clinical pain phenotypes. *MARCO* is strongly and nominally significantly positively correlated with paroxysmal pain. (B) Heatmap showing the normalised expression of these genes in Morton's neuroma participants. A cluster of participants with increased expression of *MARCO* also has increased NPSI paroxysmal pain. NPSI, Neuropathic Pain Symptom Inventory.

Given the consistent signature for immune cells and specifically the association of macrophage signatures with paroxysmal pain, we further screened our list of DEGs, specifically those in co-expression modules that correlated with symptoms in participants with Morton's neuroma or were annotated with significantly enriched GO terms associated with the immune response and the development of the nervous system. Amongst these genes, the following combination stood out as being known markers of a specific macrophage phenotype according to updated nomenclature^41^: (1) *MARCO*—macrophage receptor with collagenous structure; (2) *CD163*—typically found in resident macrophages and a high-affinity scavenger receptor for the haemoglobin-haptoglobin complex; (3) *STAB1*: Stabilin-1, a transmembrane receptor protein that is involved in angiogenesis, cell adhesion, and receptor scavenging. These 3 genes are signature markers for M(GC) macrophages, ie, those that have been stimulated with glucocorticoids.^41^ We looked for associations between these genes and symptoms in participants with Morton's neuroma. All 3 genes were positively correlated with NPSI paroxysmal pain and paraesthesia (Figs. 6A and B). *MARCO* had a strong and nominally significant correlation with paroxysmal pain (r = 0.48 95% CI [0.1, 0.76], *P*-value = 0.02). All correlations are shown in Supplemental Table A9, http://links.lww.com/PAIN/C177. Of note, the patient sample cluster from the differentially expressed genes that had the strongest expression of MARCO (Fig. 3D, cluster 3) also had significantly higher NPSI paroxysmal pain (Supplemental Figure A9, http://links.lww.com/PAIN/C177).

### 3.7. Immunohistochemistry confirms an upregulation of M(GC) MARCO+ subset of macrophages in Morton's neuroma

**Figure 7.**
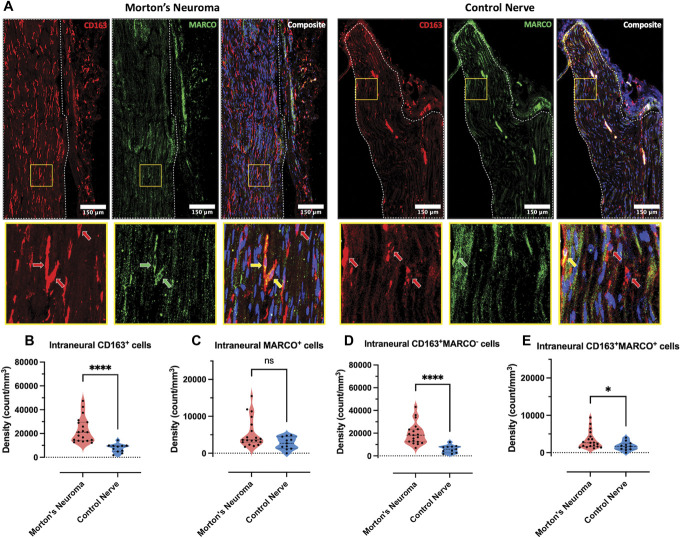
Immunohistochemistry confirms increased densities of intraneural CD163^+^, CD163^+^MARCO^+^, and CD163^+^MARCO^−^ macrophages in Morton's neuroma. (A) Representative images (stitched tile scans with 5% overlap) of nerve sections from a patient with Morton's neuroma (left) and a healthy individual (right) stained with CD163 (red), MARCO (green), and Nuclear Violet (blue). The boundaries of intraneural areas are indicated with white dotted lines. The areas within yellow squares were enlarged 21.7x and displayed below. The red, green, and yellow arrows indicate examples of CD163^+^, MARCO^+^, and CD163^+^MARCO^+^ cells, respectively. (B–E) Except for intraneural MARCO^+^ cells (C), the densities of intraneural CD163^+^ (B), CD163^+^MARCO^+^ (D), and CD163^+^MARCO^−^ cells (E) were significantly higher in Morton's neuroma compared with control nerves. Morton's neuroma, N = 19; Control Nerve, N = 11. Data are presented as medians and interquartile ranges. Mann–Whitney *U* tests, *P*-values = <0.0001 (B), 0.070 (C), <0.0001 (D) and 0.0374 (E).

We validated the RNAseq findings using immunohistochemistry of tissue sections M(GC) macrophage markers CD163 and MARCO. We confirmed higher intraneural densities of CD163^+^, CD163^+^MARCO^+^, and CD163^+^MARCO^−^ cells in Morton's neuroma compared with control nerves (Figs. 7A–E). No significant correlation was found between the intraneural densities of CD163^+^, MARCO^+^, CD163^+^MARCO^+^, or CD163^+^MARCO^−^ macrophages and patients' pain phenotype (Supplemental Table A10 and A11, http://links.lww.com/PAIN/C177).

## 4. Discussion

Using immunofluorescent staining of human Morton's neuroma and control nerves, we identified persistent demyelination, an intraneural increase in CD3^+^ T-cell densities, and an association of CD68^+^ macrophages with burning pain. Further characterisation using RNAseq revealed a large number of differentially expressed genes in injured vs control human nerves. Both GO and WGCNA analyses identified biological processes involved with a response of the immune system and inflammation and development of the nervous system (eg, myelination, axon guidance) to be overrepresented. Deconvolution analysis confirmed differences in immune cell populations with macrophage proportions not only greater in Morton's neuroma but also correlating with the severity of paroxysmal pain. Of note, we identified an overexpressed gene signature (*MARCO, CD163,* and *STAB1*) associated with a specific subset of M(GC) macrophages, with the expression of *MARCO* being positively correlated with paroxysmal pain severity. An intraneural increase in the M(GC) MARCO^+^ macrophage subset was confirmed using immunofluorescent staining. These findings implicate an ongoing role for the immune system in the context of chronic peripheral neuropathic pain in humans, with a potentially interesting role for macrophages and specifically the M(GC) MARCO^+^ macrophage subset.

Our findings highlight the role of local neuroinflammation in the context of chronic neuropathic pain in humans. Neuroinflammation and its importance in the initiation and maintenance of neuropathic pain are well established in preclinical models,^14,24^ with a particular prominence of macrophages and T cells^17,32^ but also neutrophils and mast cells. However, evidence for its role in human nerve injuries has remained largely circumstantial due to challenges of access to tissues.^59^ Most evidence to date stems from indirect methods such as systemic blood/cerebrospinal fluid analysis^26,48^ with histological analyses of nerve biopsies only performed in severe and debilitating conditions that justify nerve biopsy (eg, vasculitic neuropathies, chronic axonal neuropathies).^15,34^ Whereas these studies support the role of inflammation in severe neuropathic pain conditions in humans, our current findings suggest that milder forms of nerve injuries such as entrapment neuropathies are also associated with neuroinflammation. This is in line with our previous preclinical findings of local immune cell activation in a model of mild chronic nerve compression.^55^

Of importance, the patient population studied here had longstanding symptoms associated with nerve injury (median duration 2.5 years). Most preclinical models study the effect of neuroinflammation over relatively short periods in the order of weeks rather than years after the onset of nerve injury.^22^ These studies suggest that local immune cell densities remain high for up to 14 weeks after nerve ligation,^32^ 7 weeks after chronic constriction injury,^38^ and 12 weeks after mild nerve compression.^55^ However, the scarcity of long-term studies precludes in-depth knowledge about the exact timing of neuroinflammation persistence and its resolution.

Our data highlight that neuroinflammation continues to play a role in chronic neuropathic pain, long after the initial onset of nerve injury. For instance, macrophage subtypes enriched after acute experimental nerve injury in mouse (day 5) remained overexpressed in chronic human Morton's neuroma vs control nerves. This could be either attributed to continuing axon or myelin degeneration due to ongoing nerve compression or the failure of inflammation to resolve. Indeed, even though *CD163* (expressed on anti-inflammatory macrophages) was high at molecular and cellular levels, pro-resolution markers, such as *Arginase1* or *FoxP3*, were either absent or very lowly expressed in our dataset (Supplemental file 2, http://links.lww.com/PAIN/C176), replicating what has been reported in the mouse.^32^ The importance of resolution of neuroinflammation has gained increasing interest with studies highlighting insufficiency or dysregulation of pain-resolving immune cells and their mediators in experimental neuropathic pain.^17^ There is also emerging evidence for the pro-resolution machinery as an important player in human chronic pain persistence^47^ and resolution.^53^ A dysfunction of resolution may explain why steroid injections are only short-lasting in patients with Morton's neuroma^11^ and other entrapment neuropathies.^37^ The LR receptor analysis highlights some pathways that may be of potential therapeutic interest. For instance, the Wnt signalling pathway has an established role in pain processing,^60^ including neuropathic pain from focal nerve injury,^68^ and CD44 receptor-targeted drug strategies are currently under development for rheumatoid arthritis-related pain.^18^

Screening of differentially expressed genes associated with co-expression modules identified consistent upregulation of 3 genes (*MARCO, STAB1,* and *CD163*) associated with the subset of M(GC) glucocorticoid-induced macrophages.^41^ MARCO is a class A scavenger receptor, which has been implicated in opsonin-independent phagocytosis, cell signalling in inflammation, and macrophages that adopt a profibrotic phenotype.^23,25,42^ MARCO expression can be upregulated within hypoxic microenvironments^7^ such as found in peripheral neuropathies.^12,33^ We identified a correlation between *MARCO* expression and paroxysmal pain, although this was not replicated with immunohistological staining. It could be speculated that the stark upregulation of this type of macrophage reflects a continuing but potentially failing effort to combat the ongoing nerve compression and hypoxic intraneural environment. Alternatively, it could be that the presence of M(GC) MARCO^+^ macrophages indicates cells that are promoting fibrosis in a nonresolution immune environment,^42^ thus preventing a return to pain-free tissue homeostasis. Finally, our identification of this subset raises the question of whether steroid injections might not only be ineffective as a long-term treatment but perhaps might even promote the persistence of this potentially counter-resolution macrophage population.

In addition to a strong inflammation signature, our data also implicate processes related to the development of the nervous system. It is well established that nerve injuries are associated with demyelination, which persists to chronic stages of human entrapment neuropathies.^6,54^ The immunohistochemical experiments validated the myelination gene signature, with a stark reduction in myelin basic protein staining in Morton's neuromas.

In Morton's neuroma sections, we also observed intraneural areas that lacked axons, presumably indicating fibrotic changes (see also upregulated GO terms collagen fibril organisation and cellular response to transforming growth factor beta stimulus). Fibrotic contribution is well established in other entrapment neuropathies such as carpal tunnel syndrome, where fibrotic factors such as TGF-beta are strongly upregulated.^4,10,53^ However, early nonquantitative studies suggest that similar fibrotic areas are present in nonpainful plantar nerves.^9,40^ It could be the case that fibrosis is only proalgesic if it is accompanied by an inflammatory environment, as is the case in our Morton's neuroma samples.

Finally, while quantification of overall anti-β-tubulin III staining was not altered in Morton's neuroma compared with control nerves, it negatively correlated with pain, corroborating the findings from the WGCNA analysis where the neurogenesis module negatively correlated with paroxysmal pain.

### 4.1. Limitations

Several limitations should be considered when interpreting our findings. First, the location of control human nerves varied, with only 3 samples taken at the same anatomical site as the Morton's neuroma due to a lack of tissue availability. We deemed it more important to collect fresh rather than postmortem human nerves due to RNA degradation and age influences on peripheral nerve integrity and inflammatory processes. Differences in nerve composition (eg, motor vs sensory) may have influenced our findings. Based on the PCA plots, the different types of nerves did not cluster together, making this unlikely. The 3 control nerves collected from amputees are more similar to Morton's neuroma than the other control samples. Second, whereas our age matching was successful, the proportions of male and female participants were disparate in the 2 groups, reflecting the clinical populations of Morton's neuroma (female dominant)^31^ vs flap or amputation surgeries often secondary to trauma (male dominant). The relatively small sample size prohibited separate analyses of male and female nerve samples. Sex differences related to immune function^27^ and pain^21^ are well established and recent evidence from healthy human peripheral nerves also reported sexually dimorphic gene expressions.^51^ In this study, we considered and controlled for an additive effect of sex in Morton's neuroma vs control nerves. Sex is not one of the primary outcomes, we rather blocked for its effect on Morton's neuroma vs control nerves, so we do not report the results for this coefficient. Third, the use of human tissue comes with inherent variation (eg, chronicity, severity). While we were careful to exclude potential co-morbidities that may influence nerve health in both groups, human tissue variation is likely to mask more subtle effects. Of note, this increases confidence in the identified changes, which are likely of considerable size to survive background noise. Finally, with a limited amount of human tissue available, we focussed on validating the key findings from our bulk sequencing experiment.

## 5. Conclusions

In conclusion, our combined transcriptomic and histological findings suggest an intraneural signature related to inflammation and development of the nervous system (eg, myelination, neurogenesis) to be associated with Morton's neuroma. We identified correlations between deconvolution-derived immune cell populations, WGCNA modules, and single genes (*MARCO*) with paroxysmal pain, while histological CD68^+^ macrophage density positively correlated with burning pain. These findings implicate neuroinflammation in the chronic stage of human neuropathic pain associated with focal nerve injury. The M(GC) MARCO^+^ subset of macrophages may be of interest in the context of chronic paroxysmal pain.

## Conflict of interest statement

The authors have no conflicts of interest to declare.

## Appendix A. Supplemental digital content

Supplemental digital content associated with this article can be found online at http://links.lww.com/PAIN/C177 and http://links.lww.com/PAIN/C176.

## Supplemental video content

A video abstract associated with this article can be found on the PAIN Web site.
