# Supplementary material for: The local molecular signature of human peripheral neuropathic pain
Source: Pain. 2024 Nov 25;166(5):1143–56. doi: 10.1097/j.pain.0000000000003472 (PMC12004989; doi:10.1097/j.pain.0000000000003472)

# Supplemental Material

## Supplemental Table A1: Primary and secondary antibodies used in the study

| Primary antibody to | Host | Clonality | Isotype | Conjugated | Dilution | Catalogue no. | Company |
|---|---|---|---|---|---|---|---|
| Human myelin basic protein | Rat | Monoclonal | IgG2a | N/A | 1:500 | ab7349 | Abcam |
| Human β-tubulin III | Rabbit | Polyclonal | IgG | N/A | 1:500 | T2200 | Sigma-Aldrich |
| Human CD3 | Rabbit | Monoclonal | IgG | N/A | 1:100 | ab16669 | Abcam |
| Human CD68 | Mouse | Monoclonal | IgG1 | N/A | 1:200 | ab955 | Abcam |
| Human CD3 | Mouse | Monoclonal | IgG2a | N/A | 1:25 | ab699 | Abcam |
| Human CD4 | Rabbit | Monoclonal | IgG | N/A | 1:50 | ab133616 | Abcam |
| Human CD163 | Mouse | Monoclonal | IgG1 | N/A | 1:100 | NB-40686AF647 | Bio-techne |
| Human MARCO | Rabbit | Polyclonal | IgG | N/A | 1:50 | PA5-64134 | Invitrogen |

| Secondary antibody to | Host | Clonality | Isotype | Conjugated | Dilution | Catalogue no. | Company |
|---|---|---|---|---|---|---|---|
| Rabbit IgG (H+L) | Donkey | Polyclonal | IgG | AF546 | 1:500 | A10040 | Invitrogen |
| Rat IgG (H+L) | Goat | Polyclonal | IgG | AF488 | 1:500 | A11006 | Invitrogen |
| Mouse IgG (H+L) | Goat | Polyclonal | IgG | AF546 | 1:500 | A11003 | Invitrogen |
| Rabbit IgG (H+L) | Goat | Polyclonal | IgG | AF488 | 1:500 | A11008 | Invitrogen |

## Supplemental Table A2: Correlations between MBP or β-tubulin III (immunohistochemistry) and clinical pain phenotype

| Clinical pain scores | Corrected mean grey values of | Correlation test | r | p-value | Adjusted p-value |
|---|---|---|---|---|---|
| NPSI burning pain | Myelin basic protein | Spearman's | -0.356 | 0.161 | 0.375 |
| NPSI deep pain | Myelin basic protein | Pearson's | 0.056 | 0.831 | 0.831 |
| NPSI paroxysmal pain | Myelin basic protein | Spearman's | 0.080 | 0.759 | 0.886 |
| NPSI evoked pain | Myelin basic protein | Pearson's | -0.192 | 0.461 | 0.646 |
| NPSI paraesthesia | Myelin basic protein | Spearman's | -0.391 | 0.122 | 0.427 |
| VAS numbness (24 hours) | Myelin basic protein | Spearman's | -0.239 | 0.370 | 0.648 |
| VAS pain (24 hours) | Myelin basic protein | Pearson's | -0.488 | 0.055 | 0.387 |
| NPSI burning pain | β-tubulin III | Spearman's | -0.162 | 0.531 | 0.743 |
| NPSI deep pain | β-tubulin III | Pearson's | 0.262 | 0.310 | 0.542 |
| NPSI paroxysmal pain | β-tubulin III | Spearman's | 0.462 | 0.063 | 0.222 |
| NPSI evoked pain | β-tubulin III | Pearson's | -0.046 | 0.862 | 1.000 |
| NPSI paraesthesia | β-tubulin III | Spearman's | -0.271 | 0.292 | 0.680 |
| VAS numbness (24 hours) | β-tubulin III | Spearman's | -0.022 | 0.936 | 0.936 |
| **VAS pain (24 hours)** | **β-tubulin III** | **Pearson's** | **-0.653** | **0.006** | **0.043*** |

| Clinical pain scores | Area fractions of | Correlation test | r | p-value | Adjusted p-value |
|---|---|---|---|---|---|
| NPSI burning pain | Myelin basic protein | Spearman's | -0.269 | 0.294 | 0.515 |

| | | | | | |
|---|---|---|---|---|---|
| NPSI deep pain | Myelin basic protein | Pearson’s | 0.106 | 0.685 | 0.685 |
| NPSI paroxysmal pain | Myelin basic protein | Spearman’s | -0.167 | 0.518 | 0.605 |
| NPSI evoked pain | Myelin basic protein | Pearson’s | -0.212 | 0.414 | 0.580 |
| NPSI paraesthesia | Myelin basic protein | Spearman’s | -0.429 | 0.087 | 0.204 |
| VAS numbness (24 hours) | Myelin basic protein | Spearman’s | -0.469 | 0.069 | 0.241 |
| VAS pain (24 hours) | Myelin basic protein | Pearson’s | -0.552 | 0.027* | 0.186 |
| NPSI burning pain | β-tubulin III | Spearman’s | -0.174 | 0.502 | 0.753 |
| NPSI deep pain | β-tubulin III | Pearson’s | 0.188 | 0.469 | 1.000 |
| NPSI paroxysmal pain | β-tubulin III | Spearman’s | 0.160 | 0.537 | 0.645 |
| NPSI evoked pain | β-tubulin III | Pearson’s | -0.071 | 0.786 | 0.786 |
| NPSI paraesthesia | β-tubulin III | Spearman’s | -0.421 | 0.094 | 0.562 |
| VAS numbness (24 hours) | β-tubulin III | Spearman’s | -0.188 | 0.482 | 0.963 |
| **VAS pain (24 hours)** | **β-tubulin III** | **Pearson’s** | **-0.7096** | **0.002** | **0.015*** |

Choice of correlation tests was based on normality of data. Nominally significant correlations are in bold, FDR adjusted significant p-values are denoted by a star*

## Supplemental Table A3: Correlations between intraneural $CD3^+$ or $CD68^+$ cells (immunohistochemistry) and clinical pain phenotype

| Clinical measurement scores | The densities of | Correlation test | r | p-value | Adjusted p-value |
|---|---|---|---|---|---|
| **NPSI burning pain** | **$CD68^+$ cells** | **Spearman's** | **0.633** | **0.008** | **0.046*** |
| NPSI deep pain | $CD68^+$ cells | Pearson's | 0.142 | 0.586 | 0.703 |
| NPSI paroxysmal pain | $CD68^+$ cells | Spearman's | 0.151 | 0.560 | 0.840 |
| NPSI evoked pain | $CD68^+$ cells | Pearson's | 0.074 | 0.779 | 0.779 |
| NPSI paraesthesia | $CD68^+$ cells | Spearman's | 0.388 | 0.125 | 0.374 |
| VAS pain (24 hours) | $CD68^+$ cells | Pearson's | 0.221 | 0.411 | 0.822 |
| NPSI burning pain | $CD3^+$ cells | Spearman's | 0.466 | 0.061 | 0.366 |
| NPSI deep pain | $CD3^+$ cells | Spearman's | 0.126 | 0.629 | 0.629 |
| NPSI paroxysmal pain | $CD3^+$ cells | Spearman's | 0.166 | 0.521 | 0.626 |
| NPSI evoked pain | $CD3^+$ cells | Spearman's | 0.233 | 0.365 | 0.547 |
| NPSI paraesthesia | $CD3^+$ cells | Spearman's | 0.414 | 0.100 | 0.300 |
| VAS pain (24 hours) | $CD3^+$ cells | Spearman's | 0.297 | 0.261 | 0.523 |

Choice of correlation tests was based on normality of data. Nominally significant correlations are in bold, FDR adjusted significant p-values are denoted by a star*

## Supplemental Table A4: Correlations between intraneural $CD3^{+}CD4^{+}$ cells (immunohistochemistry) and clinical pain phenotype

| Clinical measurement scores | The densities of | Correlation test | r | p-value | Adjusted p-value |
|---|---|---|---|---|---|
| NPSI burning pain | $CD3^{+}CD4^{+}$ cells | Spearman’s | 0.075 | 0.755 | 1.000 |
| NPSI deep pain | $CD3^{+}CD4^{+}$ cells | Pearson’s | 0.065 | 0.785 | 1.000 |
| NPSI paroxysmal pain | $CD3^{+}CD4^{+}$ cells | Spearman’s | 0.450 | **0.047** | 0.279 |
| NPSI evoked pain | $CD3^{+}CD4^{+}$ cells | Pearson’s | 0.018 | 0.940 | 1.000 |
| NPSI paraesthesia | $CD3^{+}CD4^{+}$ cells | Spearman’s | 0.077 | 0.747 | 1.000 |
| VAS pain (24 hours) | $CD3^{+}CD4^{+}$ cells | Pearson’s | -0.004 | 0.988 | 0.988 |

Choice of correlation tests was based on normality of data. Nominally significant correlations are in bold.

## Supplemental Table A5: Correlations between intraneural MBP or β-tubulin III and immune cells (immunohistochemistry)

| Area fraction of | The densities of | Correlation test | r | p-value | Adjusted p-value |
|---|---|---|---|---|---|
| **Myelin basic protein** | **$CD3^+$ cells** | **Spearman's** | **-0.6594** | **0.003** | **0.020*** |
| **Myelin basic protein** | **$CD68^+$ cells** | **Pearson's** | **-0.6036** | **0.008** | **0.028*** |
| Myelin basic protein | $CD3^+CD4^+$ cells | Pearson's | -0.2268 | 0.366 | 0.426 |
| Myelin basic protein | $CD163^+$ cells | Spearman's | -0.3260 | 0.201 | 0.352 |
| Myelin basic protein | $MARCO^+$ cells | Spearman's | 0.3211 | 0.209 | 0.292 |
| Myelin basic protein | $CD163^+MARCO^+$ cells | Spearman's | 0.0956 | 0.716 | 0.716 |
| Myelin basic protein | $CD163^+MARCO^-$ cells | Pearson's | -0.4267 | 0.088 | 0.204 |
| β-tubulin III | $CD3^+$ cells | Spearman's | -0.0341 | 0.893 | 1.000 |
| β-tubulin III | $CD68^+$ cells | Pearson's | -0.0546 | 0.830 | 1.000 |
| β-tubulin III | $CD3^+CD4^+$ cells | Pearson's | 0.2162 | 0.389 | 0.907 |
| β-tubulin III | $CD163^+$ cells | Spearman's | 0.0808 | 0.7584 | 1.000 |
| β-tubulin III | $MARCO^+$ cells | Spearman's | 0.2598 | 0.3127 | 1.000 |
| β-tubulin III | $CD163^+MARCO^+$ cells | Spearman's | 0.4657 | 0.0615 | 0.431 |
| β-tubulin III | $CD163^+MARCO^-$ cells | Pearson's | -0.0114 | 0.965 | 0.965 |

Choice of correlation tests was based on normality of data. Nominally significant correlations are in bold, FDR adjusted significant p-values are denoted by a star*

**Supplemental Table A6: List of Ligand-Receptor (LR) pairs with their associated adjusted p.value (qval), the p.value for the LR pair and their associated correlation (LR.corr) of their expression across samples**

| L | R | pw.id | pw.name | qval | LR.pval | LR.corr |
|---|---|---|---|---|---|---|
| ANGPTL1 | TEK | R-HSA-202733 | Cell surface interactions at the vascular wall | 2.38E-18 | 3.44E-05 | 0.468 |
| ANGPTL1 | TEK | R-HSA-210993 | Tie2 signalling | 4.89E-06 | 3.44E-05 | 0.468 |
| ANGPTL1 | TEK | R-HSA-5673001 | RAF/MAP kinase cascade | 3.95E-12 | 3.44E-05 | 0.468 |
| CLCF1 | CRLF1 | R-HSA-447115 | Interleukin-12 family signalling | 7.59E-14 | 1.43E-10 | 0.649 |
| CLCF1 | CRLF1 | R-HSA-6783589 | Interleukin-6 family signalling | 7.78E-12 | 1.43E-10 | 0.649 |
| CLCF1 | CRLF1 | R-HSA-6788467 | IL-6-type cytokine receptor ligand interactions | 5.52E-13 | 1.43E-10 | 0.649 |
| CLCF1 | CRLF1 | R-HSA-9020956 | Interleukin-27 signalling | 5.73E-12 | 1.43E-10 | 0.649 |
| EFNA5 | EPHA4 | R-HSA-2682334 | EPH-Ephrin signalling | 8.70E-07 | 4.61E-06 | 0.263 |
| EFNA5 | EPHA4 | R-HSA-3928665 | EPH-ephrin mediated repulsion of cells | 8.70E-07 | 4.61E-06 | 0.263 |
| FN1 | CD44 | R-HSA-1474228 | Degradation of the extracellular matrix | 2.59E-10 | 5.36E-07 | 0.860 |
| FN1 | CD44 | R-HSA-202733 | Cell surface interactions at the vascular wall | 4.67E-17 | 5.36E-07 | 0.860 |
| FN1 | CD44 | R-HSA-216083 | Integrin cell surface interactions | 1.54E-07 | 5.36E-07 | 0.860 |
| HAS2 | CD44 | R-HSA-1474228 | Degradation of the extracellular matrix | 1.28E-11 | 2.32E-08 | 0.516 |
| HAS2 | CD44 | R-HSA-202733 | Cell surface interactions at the vascular wall | 3.69E-18 | 2.32E-08 | 0.516 |
| HAS2 | CD44 | R-HSA-216083 | Integrin cell surface interactions | 7.96E-09 | 2.32E-08 | 0.516 |

| | | | | | | |
|---|---|---|---|---|---|---|
| PGF | FLT1 | R-HSA-194138 | Signalling by VEGF | 4.24E-10 | 7.84E-08 | 0.525 |
| SPON2 | ITGAM | R-HSA-166016 | Toll Like Receptor 4 (TLR4) Cascade | 3.95E-12 | 1.06E-06 | 0.633 |
| SPON2 | ITGAM | R-HSA-168898 | Toll-like Receptor Cascades | 3.41E-12 | 1.06E-06 | 0.633 |
| SPON2 | ITGAM | R-HSA-202733 | Cell surface interactions at the vascular wall | 1.60E-17 | 1.06E-06 | 0.633 |
| SPON2 | ITGAM | R-HSA-216083 | Integrin cell surface interactions | 1.54E-07 | 1.06E-06 | 0.633 |
| SPON2 | ITGAM | R-HSA-6785807 | Interleukin-4 and Interleukin-13 signalling | 1.00E-14 | 1.06E-06 | 0.633 |
| VCAN | CD44 | R-HSA-1474228 | Degradation of the extracellular matrix | 1.31E-11 | 2.54E-08 | 0.953 |
| VCAN | CD44 | R-HSA-202733 | Cell surface interactions at the vascular wall | 3.69E-18 | 2.54E-08 | 0.953 |
| VCAN | CD44 | R-HSA-216083 | Integrin cell surface interactions | 8.05E-09 | 2.54E-08 | 0.953 |
| WNT4 | FZD6 | R-HSA-201681 | TCF dependent signalling in response to WNT | 6.53E-07 | 6.63E-07 | 0.509 |
| WNT4 | FZD6 | R-HSA-373080 | Class B/2 (Secretin family receptors) | 7.96E-09 | 6.63E-07 | 0.509 |
| WNT4 | FZD6 | R-HSA-3858494 | Beta-catenin independent WNT signalling | 3.22E-07 | 6.63E-07 | 0.509 |
| WNT4 | FZD6 | R-HSA-4086398 | Ca2+ pathway | 2.97E-07 | 6.63E-07 | 0.509 |
| WNT4 | FZD6 | R-HSA-4086400 | PCP/CE pathway | 7.22E-07 | 6.63E-07 | 0.509 |
| WNT4 | FZD6 | R-HSA-4641263 | Regulation of FZD by ubiquitination | 7.22E-07 | 6.63E-07 | 0.509 |
| WNT4 | FZD6 | R-HSA-4791275 | Signalling by WNT in cancer | 4.41E-07 | 6.63E-07 | 0.509 |

| | | | | | | |
|---|---|---|---|---|---|---|
| WNT4 | FZD6 | R-HSA-5340588 | RNF mutants show enhanced WNT signalling and proliferation | 6.72E-07 | 6.63E-07 | 0.509 |

**Supplemental Table A7: List of unique enriched pathways for the top significant Ligand-Receptor pairs.** Ligand (L) and Receptors (R) associated with the top enriched pathways are presented with their IDs and names

| L | R | pw.id | pw.name |
|---|---|---|---|
| {ANGPTL1;FN1;HAS2;SPON2;VCAN} | {TEK;CD44;ITGAM} | R-HSA-202733 | Cell surface interactions at the vascular wall |
| {SPON2} | {ITGAM} | R-HSA-6785807 | Interleukin-4 and Interleukin-13 signalling |
| {CLCF1} | {CRLF1} | R-HSA-447115 | Interleukin-12 family signalling |
| {CLCF1} | {CRLF1} | R-HSA-6788467 | IL-6-type cytokine receptor ligand interactions |
| {SPON2} | {ITGAM} | R-HSA-168898 | Toll-like Receptor Cascades |
| {ANGPTL1} | {TEK} | R-HSA-5673001 | RAF/MAP kinase cascade |

## Supplemental Table A8: List of genes and their relative expression values denoted as 'M1' and 'M2' macrophages in the deconvolution analysis

| LM22 gene signatures | | |
|---|---|---|
| GENE SYMBOL | LM22 Macrophages_M1 | LM22 Macrophages_M2 |
| *ABCB4* | 27.55771 | 121.432277 |
| *ACAP1* | 30.974254 | 23.05515 |
| *ACHE* | 1373.06962 | 48.585761 |
| *ACP5* | 1062.475909 | 7633.960182 |
| *AIF1* | 1611.228571 | 11610.29035 |
| *AIM2* | 2965.888162 | 148.284522 |
| *ALOX15* | 5.412216 | 430.335305 |
| *ALOX5* | 83.300898 | 248.372472 |
| *ANGPT4* | 21.218127 | 11.809701 |
| *APOBEC3A* | 4501.140479 | 148.79934 |
| *APOL3* | 11009.29392 | 437.527208 |
| *APOL6* | 967.645146 | 7.784612 |
| *AQP9* | 4309.341662 | 399.474619 |
| *ASGR1* | 25.236261 | 155.491339 |
| *ASGR2* | 26.373869 | 760.725771 |
| *ATP8B4* | 33.351428 | 1163.254876 |
| *BANK1* | 16.020271 | 29.192635 |
| *BHLHE41* | 143.868108 | 325.12977 |
| *BIRC3* | 1971.112539 | 237.501784 |
| *BPI* | 16.533686 | 25.96026 |
| *BST1* | 73.768331 | 209.464822 |
| *C5AR1* | 262.803764 | 1920.668117 |
| *CCDC102B* | 1.538368 | 2.55642 |
| *CCL13* | 182.28391 | 6581.432651 |
| *CCL14* | 1233.172359 | 2459.096418 |
| *CCL17* | 55.411581 | 620.213461 |
| *CCL18* | 1252.527933 | 12334.37123 |
| *CCL19* | 32553.88074 | 137.074204 |
| *CCL20* | 724.614868 | 12.804997 |
| *CCL22* | 464.972961 | 880.202204 |
| *CCL23* | 615.574234 | 5505.223986 |
| *CCL5* | 16862.16904 | 250.750217 |
| *CCL7* | 241.065018 | 85.74723 |
| *CCL8* | 4535.517152 | 2829.09296 |
| *CCND2* | 81.430532 | 108.189616 |
| *CCR2* | 80.9645 | 74.521205 |
| *CCR5* | 1493.347557 | 787.300008 |
| *CCR7* | 17366.91051 | 123.746312 |
| *CD180* | 120.080425 | 2787.643912 |

| | | |
|---|---|---|
| *CD1C* | 22.859693 | 167.50326 |
| *CD1D* | 373.059453 | 39.50876 |
| *CD209* | 190.945084 | 2758.297802 |
| *CD244* | 46.758659 | 96.281434 |
| *CD300A* | 211.104829 | 1737.241257 |
| *CD37* | 340.016978 | 2337.797784 |
| *CD38* | 12009.36242 | 642.610487 |
| *CD4* | 1574.336844 | 10105.34674 |
| *CD40* | 12966.60573 | 643.639203 |
| *CD7* | 13.368126 | 80.617694 |
| *CD72* | 9.588069 | 123.95208 |
| *CD80* | 3650.811207 | 160.481079 |
| *CDA* | 8.817497 | 28.81247 |
| *CFP* | 219.1912 | 1971.112539 |
| *CHI3L1* | 3395.663462 | 2050.816822 |
| *CHI3L2* | 1765.552386 | 175.383593 |
| *CHST15* | 103.487155 | 1051.6856 |
| *CLCA3P* | 0.251594 | 9.029404 |
| *CLEC10A* | 69.396554 | 5770.127086 |
| *CLEC2D* | 1465.741277 | 187.986571 |
| *CLEC4A* | 51.678716 | 6742.94324 |
| *CLEC7A* | 283.203808 | 3071.447657 |
| *CLIC2* | 1393.382865 | 2233.164343 |
| *COL8A2* | 33.843537 | 151.562355 |
| *CREB5* | 11.762749 | 59.217662 |
| *CRTAM* | 38.222697 | 85.708983 |
| *CRYBB1* | 12.127181 | 166.955398 |
| *CSF1* | 35.288498 | 23.122173 |
| *CSF3R* | 860.337222 | 737.859904 |
| *CXCL10* | 31273.67963 | 278.343404 |
| *CXCL11* | 9059.841342 | 29.35941 |
| *CXCL13* | 627.930517 | 16.760769 |
| *CXCL3* | 271.246673 | 233.330355 |
| *CXCL5* | 91.980538 | 46.38135 |
| *CXCL9* | 38944.01868 | 199.435917 |
| *CXCR5* | 212.275007 | 14.431145 |
| *CYP27A1* | 72.621008 | 75.710854 |
| *CYP27B1* | 6662.564771 | 145.701121 |
| *DACH1* | 4.273622 | 8.232486 |
| *DCSTAMP* | 171.556253 | 278.806442 |
| *DHRS11* | 18.600266 | 242.690661 |
| *DHX58* | 1394.307066 | 233.583127 |
| *DPEP2* | 16.216382 | 2410.189476 |
| *EBI3* | 20437.69231 | 311.205868 |
| *EGR2* | 23.087546 | 2175.756551 |

| | | |
|---|---|---|
| *ELANE* | 103.353302 | 206.315658 |
| *EPB41* | 7.165488 | 25.556869 |
| *FCER1A* | 56.548697 | 801.886611 |
| *FCER2* | 10.526179 | 352.928032 |
| *FCN1* | 306.829655 | 593.095894 |
| *FES* | 20.942158 | 982.200085 |
| *FOSB* | 23.170426 | 22.497432 |
| *FPR1* | 801.886611 | 408.541527 |
| *FPR2* | 274.936574 | 95.438646 |
| *FPR3* | 608.269253 | 2357.542622 |
| *FRK* | 3.762991 | 3.129846 |
| *FRMD4A* | 256.173231 | 1094.097795 |
| *FZD3* | 32.632427 | 195.35077 |
| *GGT5* | 290.215074 | 407.794036 |
| *GPC4* | 129.318831 | 145.871911 |
| *GPR1* | 0.972465 | 0.453354 |
| *GPR183* | 1631.804333 | 2474.434424 |
| *GUSBP11* | 14.61436 | 286.719961 |
| *HAL* | 90.959858 | 76.240189 |
| *HESX1* | 2324.301051 | 100.313358 |
| *HHEX* | 66.505428 | 566.324939 |
| *HK3* | 31.9761 | 159.121705 |
| *HLA-DOB* | 1573.187324 | 41.576516 |
| *HLA-DQA1* | 4169.716387 | 5225.867112 |
| *HPGDS* | 64.876999 | 138.674103 |
| *HRH1* | 145.283481 | 2195.073255 |
| *HSPA6* | 139.862714 | 207.498138 |
| *HTR2B* | 31.869533 | 216.050282 |
| *IDO1* | 26473.42255 | 91.367926 |
| *IFI44L* | 6016.733404 | 984.730251 |
| *IGHM* | 32.677241 | 26.518324 |
| *IGSF6* | 295.072409 | 1626.552661 |
| *IL12B* | 513.084829 | 18.653005 |
| *IL18RAP* | 24.975232 | 38.640956 |
| *IL1A* | 90.023426 | 27.047524 |
| *IL1B* | 675.121823 | 291.824541 |
| *IL2RA* | 3963.774053 | 146.46007 |
| *IL7R* | 2163.365124 | 85.591441 |
| *LAG3* | 1427.702674 | 97.677236 |
| *LAMP3* | 17116.19562 | 113.141386 |
| *LAT* | 162.813157 | 181.952672 |
| *LILRA2* | 317.685623 | 220.178832 |
| *LTA* | 114.104195 | 14.981975 |
| *LTC4S* | 145.924869 | 581.668952 |
| *LY9* | 28.294121 | 252.279981 |

| | | |
|---|---|---|
| *MAK* | 40.723703 | 106.229954 |
| *MAP4K1* | 31.103853 | 367.271055 |
| *MARCO* | 48.772663 | 117.151134 |
| *MEFV* | 10.082345 | 9.173433 |
| *MEP1A* | 25.482354 | 66.466162 |
| *MGAM* | 5.166818 | 5.665802 |
| *MMP9* | 2727.981464 | 16386.05747 |
| *MNDA* | 4016.523813 | 1867.617958 |
| *MS4A6A* | 402.562324 | 16167.182 |
| *MSC* | 1562.138826 | 101.484341 |
| *NCF2* | 762.035112 | 11487.88902 |
| *NFE2* | 32.428804 | 110.075989 |
| *NKG7* | 217.224577 | 6.547728 |
| *NLRP3* | 37.679105 | 322.062046 |
| *NME8* | 97.090451 | 422.959594 |
| *NPL* | 501.961637 | 4287.536344 |
| *P2RX5* | 21.687396 | 20.011502 |
| *P2RY13* | 473.333504 | 2965.888162 |
| *P2RY14* | 21.358531 | 852.556374 |
| *P2RY2* | 11.441462 | 9.46357 |
| *PADI4* | 17.759004 | 12.531005 |
| *PAQR5* | 18.280857 | 17.469058 |
| *PCDHA5* | 85.938604 | 8.255798 |
| *PIK3IP1* | 209.464822 | 1631.804333 |
| *PLA1A* | 2799.769624 | 14.403984 |
| *PLA2G7* | 1679.064856 | 1569.463396 |
| *PLEKHF1* | 73.439523 | 33.492488 |
| *PPBP* | 230.145918 | 292.991822 |
| *PPFIBP1* | 6.90617 | 11.475809 |
| *QPCT* | 113.835212 | 1278.360026 |
| *RASGRP2* | 120.640212 | 102.042176 |
| *RASGRP3* | 552.034418 | 1320.702754 |
| *REPS2* | 9.789026 | 171.230898 |
| *RNASE2* | 407.22257 | 677.713898 |
| *RRP12* | 11.728149 | 14.514662 |
| *RSAD2* | 6976.481671 | 140.167353 |
| *S100A12* | 258.300428 | 123.701236 |
| *SEC31B* | 58.50851 | 492.069202 |
| *SELL* | 234.171636 | 287.219139 |
| *SIGLEC1* | 6220.794765 | 3457.591061 |
| *SLAMF1* | 6057.915205 | 845.506509 |
| *SLAMF8* | 663.473323 | 1407.517431 |
| *SLC12A8* | 24.556462 | 25.339354 |
| *SLC2A6* | 6057.915205 | 579.733612 |
| *SLCO5A1* | 954.038207 | 39.757128 |

| | | |
|---|---|---|
| *SOCS1* | 2300.259226 | 705.135411 |
| *SPIB* | 1435.057428 | 31.210885 |
| *ST3GAL6* | 41.075062 | 192.217977 |
| *ST6GALNAC4* | 133.983253 | 243.299101 |
| *STAP1* | 372.920945 | 43.53483 |
| *STEAP4* | 22.350852 | 26.463843 |
| *TARDBPP1* | 4.672469 | 6.606825 |
| *TCF7* | 284.03179 | 43.911528 |
| *TEP1* | 69.867938 | 112.195761 |
| *TMEM255A* | 289.163953 | 167.015226 |
| *TNFAIP6* | 19190.61594 | 325.364338 |
| *TNFRSF10C* | 42.180709 | 75.929976 |
| *TNFRSF11A* | 74.820238 | 396.39139 |
| *TNFRSF4* | 1462.50333 | 441.126154 |
| *TNFSF14* | 20.454372 | 16.045444 |
| *TNIP3* | 1192.706587 | 17.3641 |
| *TREM1* | 194.725477 | 184.649791 |
| *TREM2* | 160.831659 | 3999.017687 |
| *TRIB2* | 71.304648 | 22.363741 |
| *UPK3A* | 6.818438 | 7.606821 |
| *VNN1* | 12.28114 | 120.640212 |
| *VNN2* | 6.198523 | 8.255798 |
| *WNT5B* | 11.169494 | 533.28442 |
| *ZBTB32* | 223.024334 | 14.981975 |
| *ZFP36L2* | 23.572098 | 303.576954 |
| *ZNF135* | 4.993364 | 39.757128 |

| IMMST gene signatures | | |
|---|---|---|
| GENE Symbol | IMMST macrophage_m1 | IMMST macrophage_m2 |
| *ABCA5* | 24.630897 | 24.961502 |
| *ABCB1* | 15.464132 | 13.566988 |
| *ABHD5* | 108.259229 | 85.51079 |
| *ADAM19* | 36.512586 | 33.11254 |
| *ADAMTS5* | 10.081041 | 9.472768 |
| *ADI1* | 48.616494 | 79.643998 |
| *AGPAT5* | 50.099996 | 42.497776 |
| *ALAS1* | 87.283585 | 64.273069 |
| *ANXA3* | 11.542702 | 8.375526 |
| *APOBEC3A* | 263.131611 | 37.612708 |
| *APOBEC3G* | 114.312067 | 47.078507 |
| *APOE* | 770.980636 | 468.985784 |
| *ARID4A* | 62.114806 | 77.447609 |
| *ARNT2* | 16.374199 | 9.575317 |
| *ASGR1* | 81.902272 | 96.547202 |
| *ASGR2* | 30.691987 | 66.222538 |
| *ASRGL1* | 31.970687 | 45.722372 |
| *ATP2B1* | 40.32152 | 63.084047 |
| *C1QA* | 183.454527 | 174.396667 |
| *C1QB* | 760.979895 | 415.591757 |
| *CACNA2D3* | 14.996063 | 16.45717 |
| *CALML4* | 52.953172 | 61.126032 |
| *CAMK4* | 10.568397 | 9.461182 |
| *CASP1* | 227.559694 | 128.462902 |
| *CCR2* | 19.011265 | 26.252999 |
| *CCR3* | 13.944134 | 15.410815 |
| *CD14* | 1312.160328 | 544.111938 |
| *CD163* | 323.425414 | 326.252005 |
| *CD19* | 30.032093 | 30.371927 |
| *CD1D* | 34.051541 | 24.184756 |
| *CD1E* | 22.479124 | 79.399689 |
| *CD207* | 11.735855 | 15.369275 |
| *CD209* | 180.567087 | 642.842597 |
| *CD38* | 341.629037 | 31.603112 |
| *CD3D* | 19.449839 | 16.652012 |
| *CD4* | 105.571924 | 152.733516 |
| *CD8A* | 18.086649 | 18.981875 |
| *CDC14B* | 19.040319 | 14.904207 |
| *CDC42EP4* | 118.310048 | 86.283577 |
| *CDK2AP2* | 50.337277 | 53.60228 |
| *CDKN1C* | 23.53832 | 22.066926 |

| | | |
|---|---|---|
| *CDR2L* | 23.844203 | 76.287923 |
| *CEACAM3* | 23.547452 | 22.994622 |
| *CES1* | 83.84816 | 43.647947 |
| *CFB* | 468.539663 | 64.509487 |
| *CHMP7* | 45.414453 | 42.674649 |
| *CHST7* | 129.464542 | 145.295908 |
| *CIB2* | 24.881341 | 20.905479 |
| *CLCF1* | 74.896664 | 46.968353 |
| *CLEC4E* | 58.537679 | 20.167679 |
| *CLEC5A* | 109.754963 | 39.816376 |
| *CLIC2* | 73.083241 | 124.119722 |
| *CNNM1* | 28.06154 | 29.605258 |
| *CNOT1* | 82.160237 | 94.498375 |
| *CP* | 26.831256 | 13.188198 |
| *CRISPLD2* | 78.675672 | 22.916827 |
| *CRLF2* | 17.549464 | 15.361947 |
| *CSTA* | 97.222436 | 85.573577 |
| *CXCL10* | 950.98551 | 24.653657 |
| *CXCL9* | 557.824855 | 26.517111 |
| *CYCS* | 56.353643 | 70.424246 |
| *CYP4F3* | 14.912731 | 10.663558 |
| *CYSLTR1* | 47.664642 | 52.269631 |
| *DEFB1* | 51.553472 | 16.54388 |
| *DENND3* | 75.596479 | 51.239597 |
| *DPYD* | 136.567867 | 160.421642 |
| *DUSP4* | 15.619161 | 15.879018 |
| *DYSF* | 81.318775 | 62.847265 |
| *EDN1* | 222.128369 | 41.451488 |
| *EIF1* | 1127.387628 | 1147.809467 |
| *ELSPBP1* | 12.417795 | 9.097566 |
| *EMILIN2* | 559.149007 | 596.840208 |
| *EVL* | 325.23282 | 792.525801 |
| *FCHO1* | 52.11439 | 67.194768 |
| *FCN1* | 507.278422 | 357.450773 |
| *FGFR3* | 21.19645 | 20.901608 |
| *FGR* | 230.896904 | 224.091163 |
| *FLT4* | 9.057131 | 10.352679 |
| *FMO5* | 25.903274 | 24.73977 |
| *FST* | 38.960743 | 35.174874 |
| *FSTL1* | 16.63998 | 14.025324 |
| *FUT3* | 14.130251 | 15.878089 |
| *FXYD6* | 71.340161 | 36.164957 |
| *GAS7* | 43.743887 | 56.949024 |
| *GATA2* | 13.724963 | 11.863059 |
| *GBP1* | 896.719452 | 71.273239 |

| | | |
|---|---|---|
| *GCH1* | 137.261667 | 19.51714 |
| *GFOD1* | 85.044203 | 121.400191 |
| *GIMAP4* | 396.81946 | 166.154557 |
| *GLRA1* | 10.249014 | 10.443668 |
| *GPNMB* | 1031.714985 | 1121.365578 |
| *HAGH* | 157.716157 | 156.487197 |
| *HAVCR1* | 7.744264 | 7.164532 |
| *HBD* | 12.990704 | 12.819894 |
| *HCK* | 534.413194 | 408.408765 |
| *HDC* | 10.868859 | 12.853772 |
| *HOMER2* | 17.154798 | 124.184141 |
| *HPSE* | 52.394734 | 33.544332 |
| *HTRA1* | 42.297892 | 38.709417 |
| *IFI27* | 605.424453 | 34.217672 |
| *IFNB1* | 11.83225 | 8.995349 |
| *IFT20* | 42.159323 | 65.158177 |
| *IL15RA* | 66.526337 | 17.317901 |
| *IL1R2* | 31.41673 | 54.540967 |
| *IL2RA* | 68.765118 | 21.081191 |
| *IL32* | 69.334626 | 21.187101 |
| *IL5RA* | 23.854369 | 22.964176 |
| *IL6ST* | 36.237525 | 34.355542 |
| *ING2* | 108.432819 | 121.012868 |
| *IRS1* | 13.367414 | 12.076048 |
| *JRKL* | 29.091427 | 28.107682 |
| *KCNJ15* | 19.471191 | 13.970518 |
| *KIF1A* | 10.596884 | 9.506352 |
| *KIF22* | 22.122568 | 20.208357 |
| *KLHL18* | 52.583207 | 51.647409 |
| *KLRC3* | 10.861977 | 9.579869 |
| *KLRF1* | 21.005789 | 25.999136 |
| *KRT5* | 10.378597 | 10.101124 |
| *KSR1* | 23.544305 | 18.866856 |
| *LAIR1* | 413.762792 | 302.280213 |
| *LAMP3* | 133.221992 | 47.03768 |
| *LILRA4* | 33.753721 | 41.414371 |
| *LILRA5* | 111.697649 | 44.101029 |
| *LILRB1* | 283.362864 | 206.378371 |
| *LIMA1* | 74.208216 | 149.422444 |
| *LIMK2* | 54.284855 | 21.660486 |
| *LIPF* | 23.402844 | 20.990309 |
| *LRP5L* | 61.170911 | 74.419544 |
| *LRRC8D* | 81.105576 | 89.427665 |
| *LSM4* | 192.355209 | 205.887501 |
| *MAG* | 14.515489 | 14.017755 |

| | | |
|---|---|---|
| *MAL* | 16.023397 | 24.780659 |
| *MAOA* | 70.411593 | 476.221065 |
| *MAPK7* | 51.413285 | 48.983391 |
| *MAT2B* | 130.12854 | 175.130871 |
| *MEST* | 11.403056 | 11.188716 |
| *MMP25* | 49.681395 | 41.162143 |
| *MMP8* | 7.394264 | 6.948941 |
| *MMP9* | 3825.404778 | 2402.56263 |
| *MOCS3* | 25.069081 | 27.340857 |
| *MPO* | 45.855922 | 42.880359 |
| *MPPED2* | 6.65776 | 6.769345 |
| *MRPL3* | 111.359692 | 165.734104 |
| *MRPL4* | 43.657143 | 45.236235 |
| *MS4A1* | 12.435397 | 13.626376 |
| *MS4A6A* | 84.453534 | 675.750915 |
| *MT1X* | 624.849502 | 146.602072 |
| *MTMR11* | 45.061238 | 33.356051 |
| *MTSS1* | 53.515305 | 86.251774 |
| *MUC1* | 61.029106 | 27.572684 |
| *MYLIP* | 105.479133 | 111.74344 |
| *NAGA* | 82.301948 | 150.593305 |
| *NBN* | 107.383916 | 69.558193 |
| *NBR1* | 20.301084 | 32.997768 |
| *NDRG2* | 48.461579 | 52.430672 |
| *NOTCH4* | 25.50794 | 16.457332 |
| *NPEPPS* | 59.871167 | 74.879931 |
| *NR2E3* | 10.350706 | 10.206289 |
| *NR4A2* | 49.373216 | 34.249654 |
| *NRG1* | 11.224971 | 11.929372 |
| *NRGN* | 24.088339 | 47.573654 |
| *NUDT1* | 40.586782 | 64.728852 |
| *NUDT18* | 105.166233 | 81.773497 |
| *NXT1* | 187.901648 | 191.35917 |
| *NXT2* | 94.351477 | 85.265605 |
| *OLFM1* | 23.836636 | 21.128798 |
| *OR2F1* | 9.727019 | 9.947481 |
| *OR2J2* | 8.206225 | 8.262144 |
| *ORM1* | 71.607499 | 15.634066 |
| *OSBPL10* | 16.201821 | 23.093357 |
| *PALLD* | 64.220082 | 322.839532 |
| *PANX1* | 93.926979 | 38.536156 |
| *PAX5* | 12.432153 | 10.260643 |
| *PCGF2* | 54.521158 | 38.416585 |
| *PDGFB* | 31.85218 | 39.577951 |
| *PDK4* | 32.039133 | 41.872915 |

| | | |
|---|---|---|
| *PGLYRP1* | 16.973305 | 17.564939 |
| *PI3* | 18.590268 | 12.491856 |
| *PIK3CG* | 85.420994 | 81.712739 |
| *PLAT* | 14.389554 | 11.157077 |
| *POU2AF1* | 13.441854 | 16.72148 |
| *PPA1* | 883.864571 | 284.584418 |
| *PROM1* | 8.904687 | 12.974965 |
| *PSAT1* | 27.617124 | 24.651705 |
| *PTPN13* | 10.596448 | 11.97721 |
| *PTTG2* | 47.705921 | 43.386717 |
| *QPRT* | 47.82859 | 262.219569 |
| *RAB9A* | 334.077305 | 314.48347 |
| *RAMP1* | 22.138471 | 192.592609 |
| *RETN* | 15.702533 | 13.807824 |
| *RNASE1* | 98.189993 | 293.296595 |
| *RNASE4* | 40.494257 | 42.010005 |
| *RNF122* | 33.337548 | 31.926228 |
| *RPL7* | 121.95721 | 185.192881 |
| *RRAS* | 271.274825 | 203.627594 |
| *S100A12* | 31.095649 | 13.012204 |
| *S100B* | 10.358643 | 11.899717 |
| *S100P* | 27.529592 | 20.798301 |
| *SCRN1* | 73.293637 | 90.385334 |
| *SERPINF2* | 22.968984 | 27.100463 |
| *SETBP1* | 17.205978 | 20.812313 |
| *SF3A3* | 146.546483 | 184.997574 |
| *SFTPD* | 11.760854 | 17.679207 |
| *SFXN3* | 49.675081 | 52.549458 |
| *SH3BP2* | 29.562566 | 28.184269 |
| *SIDT1* | 14.048635 | 13.890627 |
| *SIGLEC6* | 17.207657 | 20.742556 |
| *SLC12A3* | 11.621312 | 11.100721 |
| *SLC15A3* | 1508.414675 | 1224.507916 |
| *SLC17A5* | 23.011273 | 37.038666 |
| *SLC1A4* | 117.395652 | 53.088537 |
| *SLC4A1AP* | 73.529014 | 71.101557 |
| *SLC6A13* | 41.57943 | 36.114688 |
| *SLC7A7* | 1257.300977 | 647.250127 |
| *SLC9A3R1* | 115.444262 | 97.944347 |
| *SLCO2B1* | 144.379303 | 353.058568 |
| *SMARCD3* | 38.19507 | 36.757718 |
| *SOCS2* | 51.89447 | 30.662962 |
| *STAB2* | 9.210147 | 7.147536 |
| *STEAP4* | 12.781317 | 13.029812 |
| *SYNE1* | 15.641658 | 19.558229 |

| | | |
|---|---|---|
| *TAGLN* | 47.473968 | 43.976212 |
| *TBC1D8* | 89.97544 | 75.585088 |
| *TCL1A* | 14.626984 | 13.342963 |
| *TFEC* | 44.374457 | 53.039233 |
| *TLL1* | 7.336204 | 7.513473 |
| *TLR5* | 42.20772 | 96.719577 |
| *TLR8* | 160.354789 | 88.426538 |
| *TM4SF5* | 7.056287 | 7.145807 |
| *TMC6* | 141.064862 | 189.397456 |
| *TNFRSF13B* | 16.406338 | 15.627486 |
| *TNFRSF25* | 13.7575 | 18.546814 |
| *TNNI2* | 36.851402 | 42.340209 |
| *TOMM22* | 126.506229 | 134.698281 |
| *TRAF3IP2* | 53.077523 | 29.147313 |
| *TRAT1* | 13.846022 | 10.402862 |
| *TREM1* | 60.239799 | 36.983424 |
| *TRIB1* | 351.065142 | 440.992267 |
| *TSPAN7* | 10.389817 | 42.686842 |
| *TUBB6* | 174.303105 | 203.20431 |
| *TULP2* | 8.486431 | 10.022635 |
| *UBE2J1* | 225.380084 | 187.877026 |
| *ULK2* | 43.445832 | 37.428174 |
| *WEE1* | 29.493918 | 22.905962 |
| *ZC3H12A* | 324.661191 | 132.978126 |
| *ZDHHC13* | 44.326729 | 37.377875 |
| *ZNF180* | 11.159899 | 10.36369 |
| *ZNF189* | 51.875213 | 47.874599 |
| *ZNF34* | 42.127202 | 48.465928 |
| *ZNF593* | 113.078329 | 95.599543 |

**Supplemental Table A9: Correlations of top enriched GO terms and selected gene markers of M(GC) MARCO$^{+}$ macrophages with clinical pain phenotype**

| Top enriched GO term | NPSI burning | NPSI deep | NPSI paroxysmal | NPSI evoked | NPSI paraesthesia | NPSI total | VAS pain 24H | VAS numbness 24H |
|---|---|---|---|---|---|---|---|---|
| immune response | 0.07 | 0.28 | 0.31 | 0.32 | -0.01 | 0.21 | 0.03 | -0.06 |
| collagen metabolic process | 0.15 | 0.14 | 0.37 | 0.1 | 0.01 | 0.15 | 0.09 | -0.12 |
| defence response | 0.02 | 0.11 | **0.66*** | 0.04 | 0.24 | 0.25 | 0.09 | -0.16 |
| muscle system process | 0.18 | -0.14 | -0.29 | **-0.44*** | -0.3 | -0.29 | -0.04 | -0.1 |
| carbohydrate transmembrane transport | -0.17 | 0.32 | 0.17 | 0.04 | -0.08 | 0.11 | -0.22 | 0.07 |
| regulation of neuron death | 0.15 | 0.01 | -0.38 | -0.19 | -0.31 | -0.2 | -0.03 | -0.01 |
| mitochondrion organization | -0.19 | -0.06 | 0.12 | 0.23 | -0.05 | 0.02 | -0.15 | 0.24 |
| neurogenesis | -0.12 | 0.09 | **-0.54*** | 0.32 | -0.13 | -0.05 | -0.12 | 0.25 |
| reproductive behaviour | -0.18 | -0.02 | -0.35 | 0.2 | -0.05 | -0.07 | -0.17 | 0.11 |
| Genes not belonging in any other module | 0.05 | 0.24 | 0.36 | 0.36 | 0.06 | 0.26 | 0.07 | 0.07 |
| **Selected gene markers of MARCO+ macrophage subset** | | | | | | | | |
| **Gene** | | | | | | | | |
| *STAB1* | -0.01 | 0.09 | 0.23 | -0.17 | 014 | 0.04 | -0.11 | -0.06 |
| *MARCO* | -0.23 | -0.03 | **0.48** | -0.05 | 0.16 | 0.07 | -0.1 | -0.07 |
| *CD163* | -0.05 | 0.05 | 0.19 | -0.35 | 0.18 | 0 | -0.12 | -0.15 |

Nominally significant correlations (p value < 0.05) are in bold, FDR adjusted significant (FDR <0.05) are denoted by a star*

## Supplemental Table A10: Correlations between intraneural $CD163^{+}$ or $MARCO^{+}$ cells (immunohistochemistry) and clinical pain phenotype

| Clinical measurement scores | The densities of | Correlation test | r | p-value | Adjusted p-value |
|---|---|---|---|---|---|
| NPSI burning pain | $CD163^{+}$ cells | Spearman's | -0.193 | 0.444 | 0.665 |
| NPSI deep pain | $CD163^{+}$ cells | Spearman's | -0.019 | 0.941 | 1.000 |
| NPSI paroxysmal pain | $CD163^{+}$ cells | Spearman's | 0.321 | 0.194 | 1.000 |
| NPSI evoked pain | $CD163^{+}$ cells | Spearman's | 0.196 | 0.435 | 0.870 |
| NPSI paraesthesia | $CD163^{+}$ cells | Spearman's | 0.263 | 0.291 | 0.874 |
| VAS pain (24 hours) | $CD163^{+}$ cells | Spearman's | 0.012 | 0.996 | 0.996 |
| NPSI burning pain | $MARCO^{+}$ cells | Spearman's | -0.295 | 0.234 | 0.702 |
| NPSI deep pain | $MARCO^{+}$ cells | Spearman's | 0.192 | 0.445 | 0.668 |
| NPSI paroxysmal pain | $MARCO^{+}$ cells | Spearman's | 0.312 | 0.207 | 1.000 |
| NPSI evoked pain | $MARCO^{+}$ cells | Spearman's | 0.139 | 0.583 | 0.699 |
| NPSI paraesthesia | $MARCO^{+}$ cells | Spearman's | 0.295 | 0.235 | 0.471 |
| VAS pain (24 hours) | $MARCO^{+}$ cells | Spearman's | -0.081 | 0.758 | 0.758 |

Choice of correlation tests was based on normality of data

**Supplemental Table A11: Correlations between intraneural $CD163^{+}MARCO^{+}$ or $CD163^{+}MARCO^{-}$ cells (immunohistochemistry) and clinical pain phenotype**

| Clinical measurement scores | The densities of | Correlation test | r | p-value | Adjusted p-value |
|---|---|---|---|---|---|
| NPSI burning pain | $CD163^{+}MARCO^{+}$ cells | Spearman's | -0.211 | 0.402 | 1.000 |
| NPSI deep pain | $CD163^{+}MARCO^{+}$ cells | Spearman's | 0.139 | 0.582 | 0.873 |
| NPSI paroxysmal pain | $CD163^{+}MARCO^{+}$ cells | Spearman's | 0.204 | 0.418 | 1.000 |
| NPSI evoked pain | $CD163^{+}MARCO^{+}$ cells | Spearman's | 0.089 | 0.726 | 0.726 |
| NPSI paraesthesia | $CD163^{+}MARCO^{+}$ cells | Spearman's | 0.193 | 0.444 | 0.888 |
| VAS pain (24 hours) | $CD163^{+}MARCO^{+}$ cells | Spearman's | -0.108 | 0.679 | 0.815 |
| NPSI burning pain | $CD163^{+}MARCO^{-}$ cells | Spearman's | -0.138 | 0.584 | 0.877 |
| NPSI deep pain | $CD163^{+}MARCO^{-}$ cells | Pearson's | 0.035 | 0.890 | 0.890 |
| NPSI paroxysmal pain | $CD163^{+}MARCO^{-}$ cells | Spearman's | 0.424 | 0.079 | 0.476 |
| NPSI evoked pain | $CD163^{+}MARCO^{-}$ cells | Pearson's | -0.061 | 0.810 | 0.972 |
| NPSI paraesthesia | $CD163^{+}MARCO^{-}$ cells | Spearman's | 0.272 | 0.275 | 0.826 |
| VAS pain (24 hours) | $CD163^{+}MARCO^{-}$ cells | Pearson's | -0.191 | 0.462 | 0.923 |

Choice of correlation tests was based on normality of data

**Supplemental Figure A1: Location of Morton's neuroma sample collection.**

Two samples (pink) were taken just proximal to the bifurcation of the plantar digital nerve and used for molecular and cellular analyses. These samples represent the location of the Morton's neuroma under the transverse metatarsal plantar ligament. The distal branches (green) were stored but not used for analyses in this paper.

Plantar digital nerve

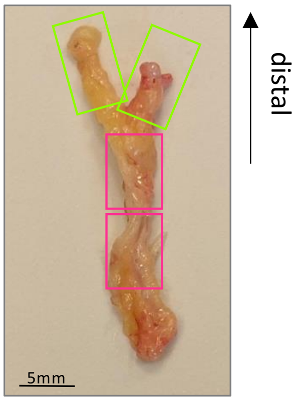

**Supplemental Figure A2: Validity of antibodies.**

(A) Immunofluorescent staining demonstrating validity of immune cell specific antibodies using human tonsillar tissue as a positive control. (B) Non-primary antibody staining used as a negative control to confirm detection of antigens by primary antibodies in the absence of secondary antibody autofluorescence in human tonsils and nerves.

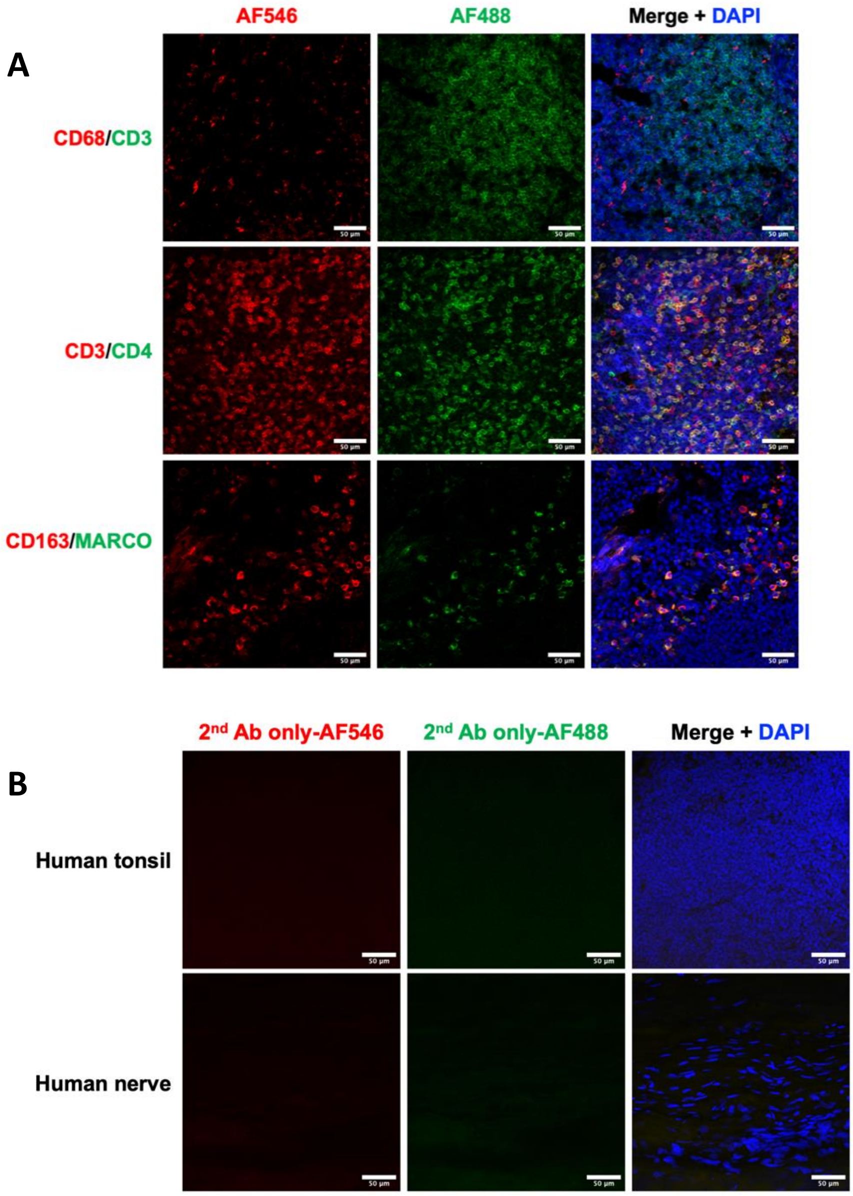

**Supplemental Figure A3: Comparison of self-reported neuropathic pain profiles among different peripheral neuropathy cohorts.** Graphs show results from the Neuropathic Pain Symptom Inventory including its subscales for the Morton's neuroma, 'sciatica', carpal tunnel syndrome (CTS) and diabetic neuropathy cohorts. Violin plots depicting median and quartiles as well as single data points. P-values derived from univariate ANCOVA corrected for age and gender, adjusted for multiple comparisons.

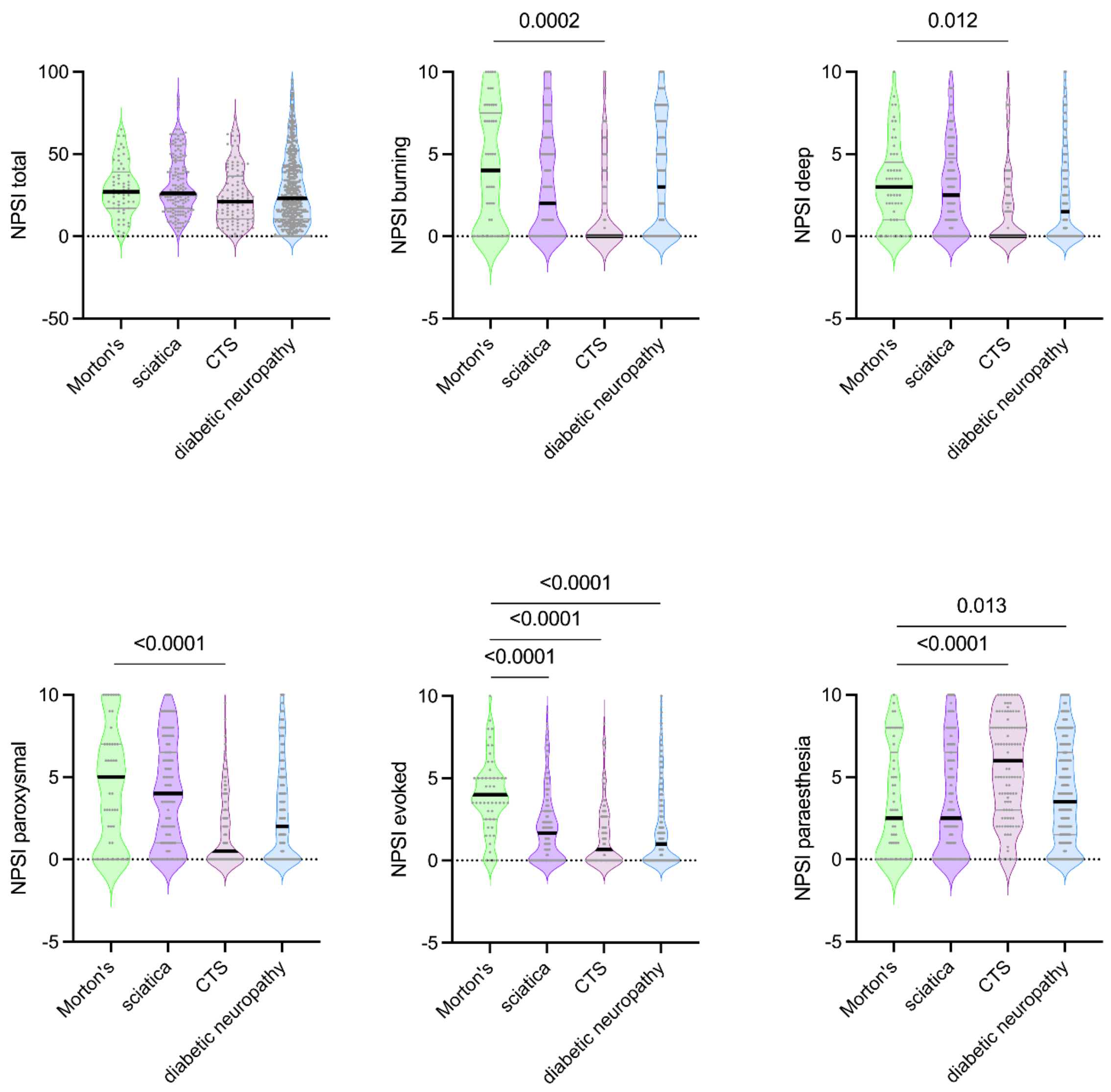

**Supplemental Figure A4: The densities of intraneural CD3⁺CD4⁺ cells are comparable between Morton's neuroma and healthy control nerves. (A)** Representative images (stitched tile scans with 5% overlap) of nerve sections from a patient with Morton's neuroma (left) and a healthy individual stained with anti-CD3 (red), CD4 (green) antibodies and DAPI (blue). The boundaries of intraneural areas are indicated with white dotted lines. The areas within yellow squares were enlarged 11.5 times and displayed below. The red, green and yellow arrows indicate examples of $CD3^+$, $CD4^+$ and $CD3^+CD4^+$ cells respectively. **(B)** The density of intraneural $CD3^+CD4^+$ was comparable between groups. Morton's Neuroma, N=21; Control Nerve, N=11. Data are presented as means and standard deviations. Student's t tests, ns: p =0.7147.

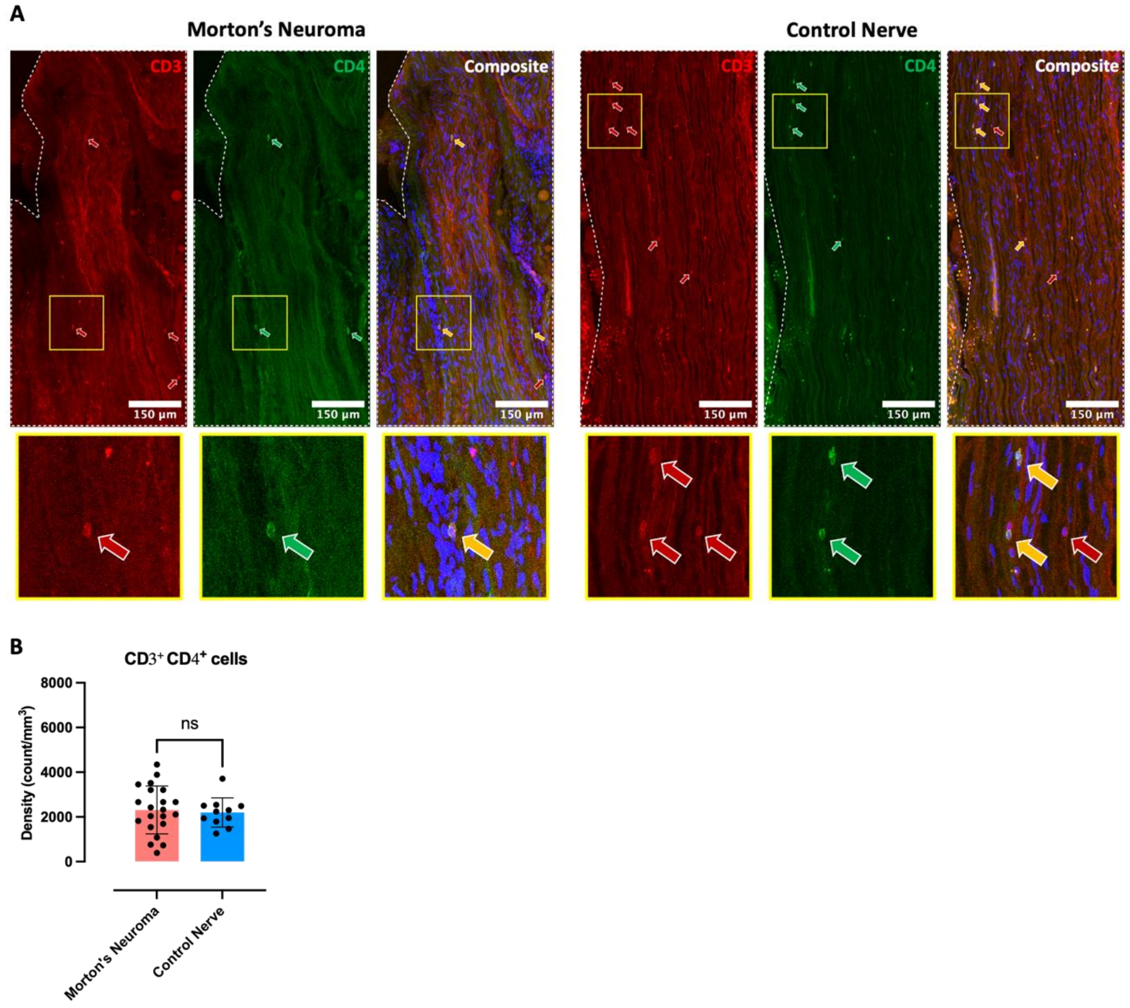

**Supplemental Figure A5: The area fractions of intraneural MBP significantly negatively correlate with the densities of intraneural $CD3^+$ and $CD68^+$ cells.** Negative correlations of area fractions of intraneural MBP with densities of **(A)** intraneural $CD3^+$ and **(B)** $CD68^+$ cells. The type of correlation test based on normality of data, r and FDR-adjusted p-value are indicated in each plot. N=18, *: $p < 0.05$

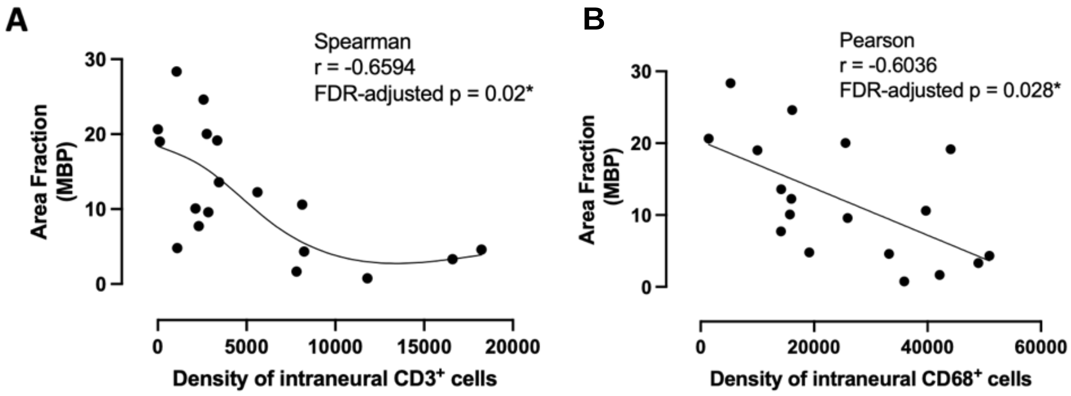

**Supplemental Figure A6: Axonally transcribed genes.**

(A)Heatmap showing the normalised expression of axonally transcribed genes. Variance stabilised gene counts were z-transformed and samples were clustered based on the expression of axonally transcribed genes using hierarchical clustering with the ward method. The condition, sex and age of samples are colour coded. (B) Signature of axonally transcribed genes in Morton’s neuroma versus control nerves. The signature is calculated as the first principal component of the axonally transcribed genes and the respective coordinates of the sample projections on the principal component.

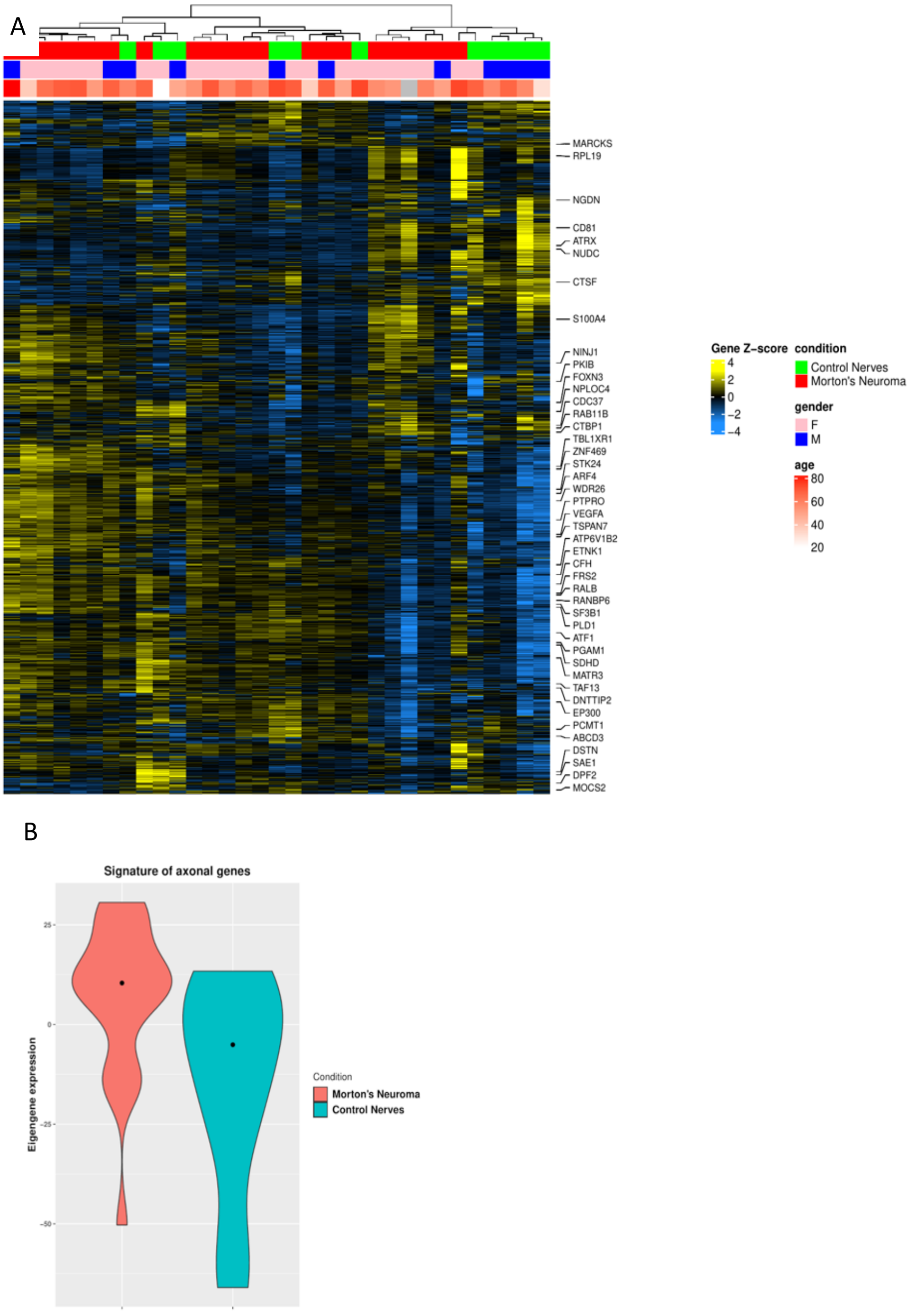

**Supplemental Figure A7: Ligand-Receptor pairs of genes and their associated pathways.** (A) Alluvial plot showing interaction between Ligands and Receptors and their associated pathways. (B) Chord plot showing the genes associated with the top enriched pathways. The correlation between the expression of the Ligand and Receptor is colour coded.

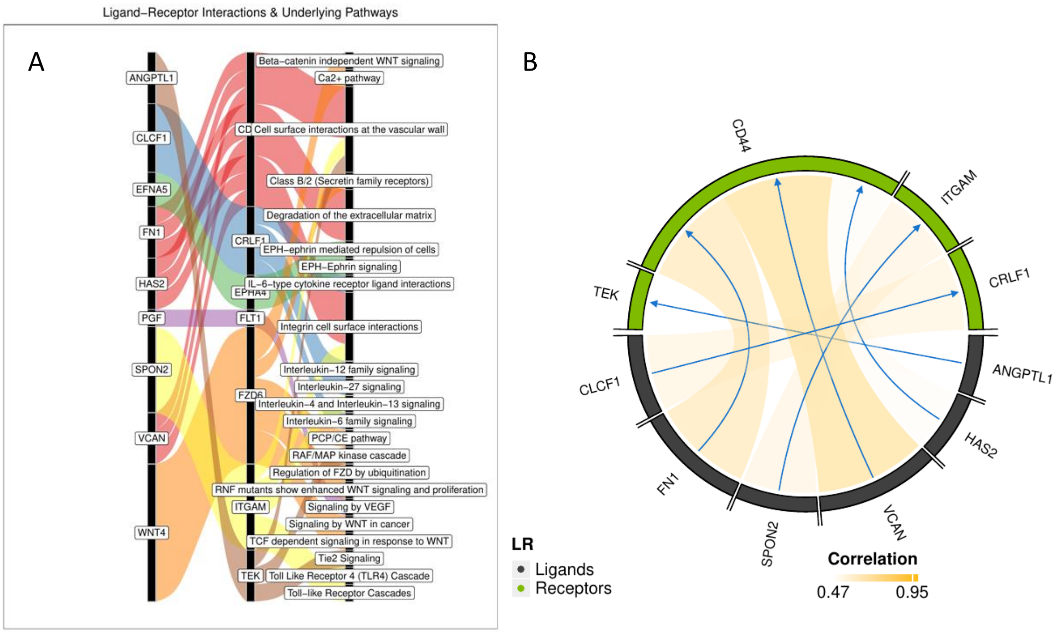

**Supplemental Figure A8: Boxplots showing inferred cell proportions for the subtypes of macrophages (0-6) identified by Ydens et al 2020[65].** StSt stands for Steady State, D1 for Day 1 post sciatic nerve crush, D5 for Day 5 post sciatic nerve crush.

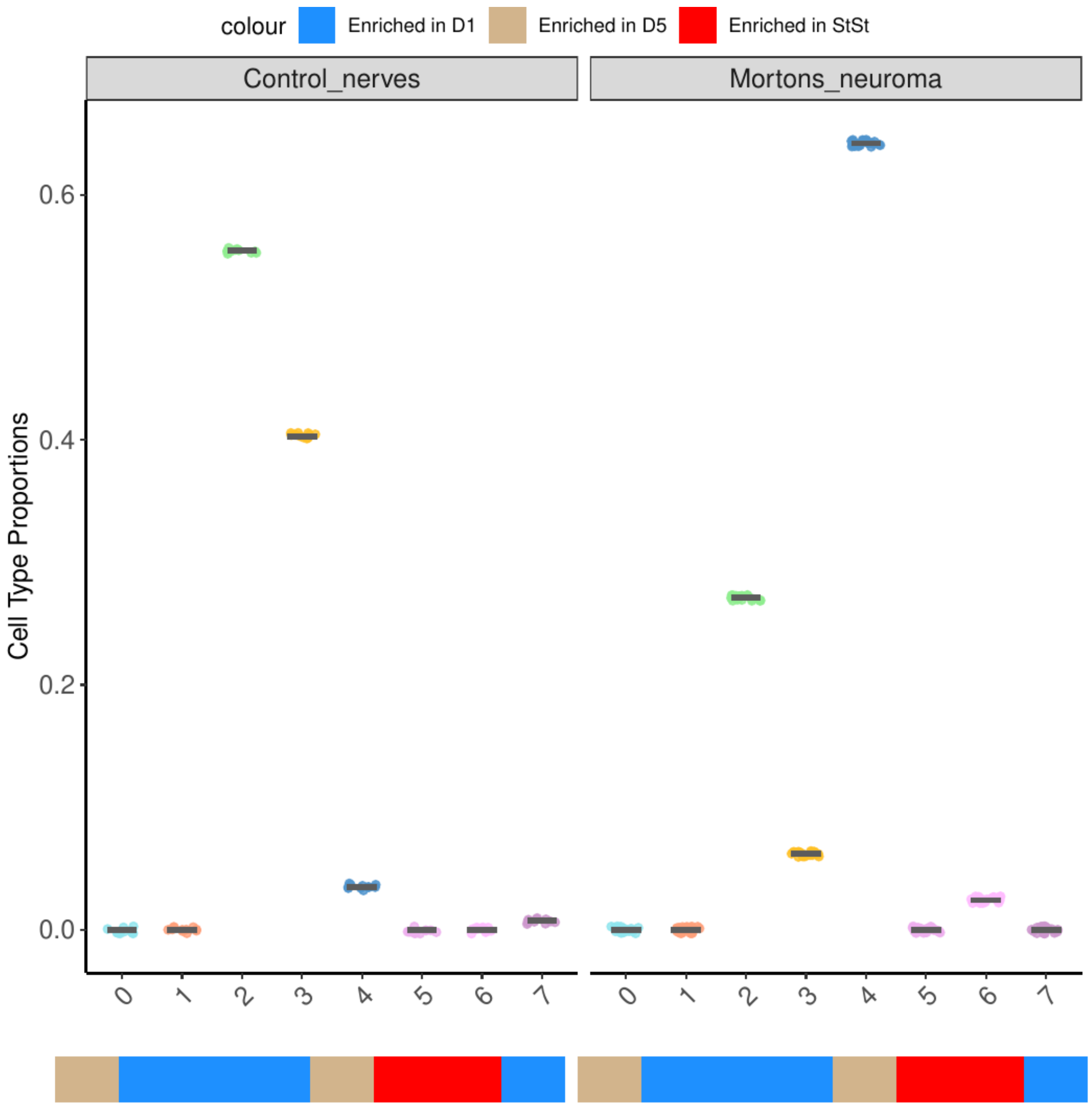

**Supplemental Figure A9: Associations of differential gene expression cluster membership with clinical phenotype.**

Tests for associations between cluster assignment identified in differential gene expression heatmap (Figure 3D) with age, symptom duration, VAS pain in the last 24h, VAS numbness in the last 24h and NPSI subscores (Kruskal-Wallis tests followed by Wilcoxon post-hoc tests). There was a significant difference in NPSI paroxysmal pain (p.value = 0.0078) between clusters 2 and 3 (Morton's Neuroma clusters). Cluster 3, in which MARCO is expressed higher (Figure 3D) is also the cluster showing higher NPSI paroxysmal pain.

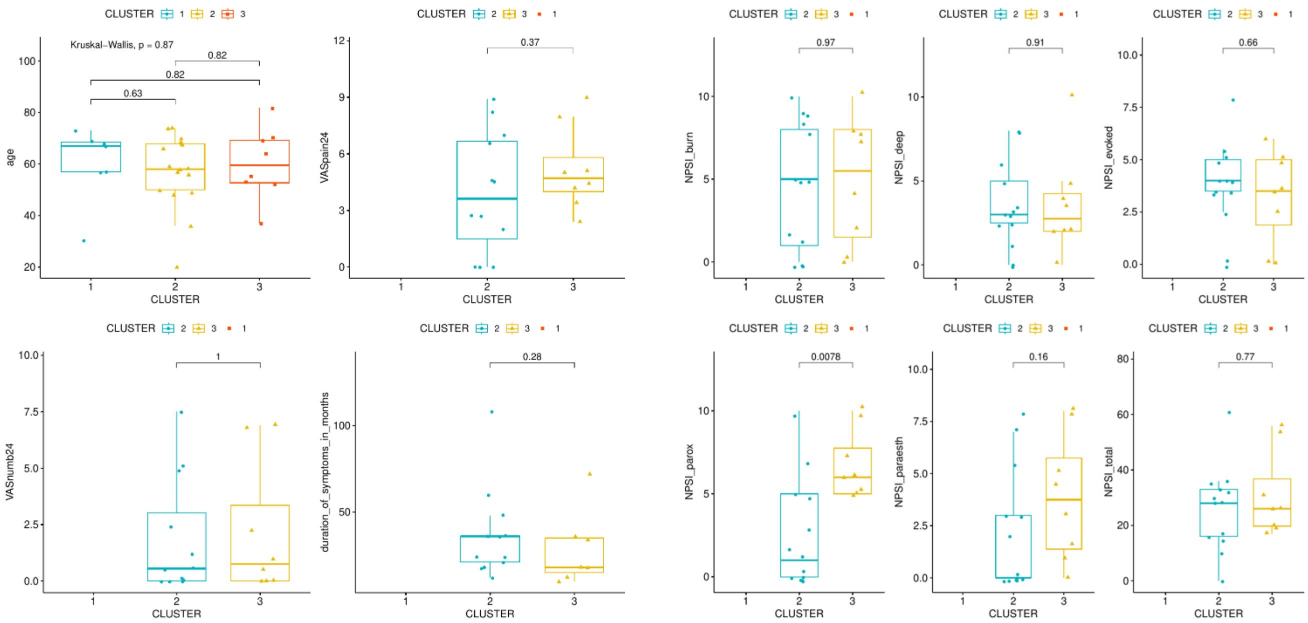

Supplement: Supplementary file 2 [file jop-166-1143-s002.pdf]
